# Systemic drivers of toxic food adulteration: lead chromate in turmeric across eastern India

**DOI:** 10.1038/s41538-026-00867-8

**Published:** 2026-05-06

**Authors:** Jenna E. Forsyth, Manu Sinha, Amogh Bandekar, Dinsha Mistree, Manoj Parida, Emily Nash, Lavanya Nambiar, Christlee Elmera, Stephen P. Luby

**Affiliations:** 1 School of Medicine, Stanford University, Stanford, CA, USA; 2 King Center on Global Development, Stanford, CA, USA; 3 Frameworks, Bhopal, India; 4 Hoover Institution, Stanford University, Stanford, CA, USA; 5 DCOR, New Delhi, India; 6 Pure Earth, New York City, NY, USA

**Keywords:** Health care, Medical research

## Abstract

Food adulteration poses a growing threat to the integrity of food systems and public health globally. This study investigated turmeric adulteration with lead chromate across five eastern Indian states through a combination of sample analysis and qualitative supply chain assessments. We collected 503 turmeric samples from 34 cities and conducted 128 stakeholder interviews between 2021-2023. In total, 30% of turmeric samples exceeded India’s permissible lead limit of 10 µg g⁻¹, with the highest levels in the state of Bihar (geometric mean: 48 µg g⁻¹, maximum: 6,416 µg g⁻¹). Adulteration primarily served to enhance turmeric root color and extend shelf life. Although regulations prohibit lead in turmeric, enforcement has been inconsistent—hindered by limited capacity and the prioritization of more immediate or visible issues. Using population-level exposure modeling, we estimate that halting the practice of turmeric adulteration with lead chromate could increase child IQ by up to 2.3 points, resulting in future income gains of US$ 239 million to 1.6 billion annually in Bihar alone. If cardiovascular disease mortality reductions are included, there would be an additional benefit of approximately US$ 430 million to 2.8 billion per year. Our findings underscore the urgent need for strengthened governance and targeted supply chain interventions.

## Introduction

Turmeric is a widely consumed spice across South Asia, a region that accounts for nearly a quarter of the global population^1,2^. India produces about 80% of the world’s turmeric supply^3^, and global demand continues to rise, driven in part by growing recognition of turmeric’s anti-inflammatory and medicinal properties^4^. Turmeric is also among the spices most vulnerable to adulteration^5^. Because spices are typically traded in their processed forms and incorporated into other foods, adulteration can be hidden without consumers knowing. Among the most harmful adulterants are lead chromate pigments, added to spices to improve visual appeal and profitability despite their well-documented toxicity^6^.

Lead is a potent toxicant harming nearly every system in the body, with adverse impacts for children and adults^7,8^. Early childhood lead exposure has adverse long-term impacts on brain development and intellectual capacity^8^. In adulthood, lead exposure is associated with increased risks of hypertension and cardiovascular disease and mortality^9^. Globally, the combined economic losses from lead-related IQ deficits and cardiovascular disease and mortality are estimated to be upwards of US$ 6 trillion per year^10^. In India alone in 2019, lead exposure was estimated to cost US$ 259 billion or 9% of GDP^10^. While blood lead levels fell precipitously after phasing out lead from gasoline in higher income countries, in other parts of the world, particularly in South Asia, blood lead levels have remained elevated decades after lead was removed from gasoline^11^.

Adulterated turmeric has been identified as a source of lead poisoning globally^12,13^. Although there is no safe level of lead consumption, international safety standards for lead in turmeric vary and are often limited. The European Union has set a maximum limit for lead in root and rhizome spices at 1.5 µg g⁻¹, compared with 2.5 µg g⁻¹ in Bangladesh and 10 µg g⁻¹ in India; thus, turmeric failing to meet stricter international standards may still be legally sold domestically^14–16^. In a recent study assessing turmeric lead levels across South Asia (India, Nepal, Pakistan, and Sri Lanka), the Indian state of Bihar was identified as a hotspot of lead chromate adulteration, where 92% of samples exceeded the limit^17^. Another study assessed child lead exposure at two sites in Bihar—one located near, and one far from, a known lead-polluting battery recycling industry^18^. Surprisingly, the children’s blood lead levels were elevated in both sites, indicating additional sources of lead exposure and hypothesized to be related to the consumption of lead-tainted spices. Turmeric sampled from the children’s homes had the highest lead levels, up to 5,000 µg g⁻¹. A subsequent state-wide study determined that elevated blood lead levels among children were associated with spice lead levels (aOR 1.35, 1.17-1.53)^19^.

In this study, we assessed the adulteration of turmeric with lead chromate in Bihar and four neighboring states to determine the geographic extent of the problem. The study team collected 503 turmeric samples from retail and wholesale bazaars in 34 of the largest cities in the five states during the summer season in 2021 and 2023. Turmeric lead levels were analyzed and compared by type, state of harvest and state of sale. We modeled the annual economic benefit of halting lead chromate turmeric adulteration in Bihar and conducted 128 qualitative interviews with stakeholders across the supply chain to identify patterns of and incentives for lead chromate turmeric adulteration and barriers to regulatory enforcement.

## Results

### Supply chain structure and key actors

Key turmeric supply chain actors included farmers, processors, millers, traders, wholesalers and retailers. Farmers primarily produced, cleaned, boiled, and dried the turmeric. Traders-cum-processors then purchased, transported, graded and polished the roots before handing over to millers. Millers ground turmeric root into powder, along with other spice types and commodities. Wholesale vendors purchased turmeric directly from traders and millers or indirectly using intermediaries known as commission agents, then sold turmeric in bulk. Retail vendors purchased turmeric from wholesale vendors and sold smaller quantities at shops known as kirana shops. (Supplementary Figs. 1 and 2; Supplementary Table 1).

Across India, the major turmeric-producing states include Maharashtra, Tamil Nadu, Telangana, and Madhya Pradesh and Karnataka^20^. The majority of turmeric traded in Bihar reportedly came from Tamil Nadu (Salem and Erode), Maharashtra (Sangli), Telangana (Nizamabad), and Karnataka (Mysore). Less than 15% of the turmeric being traded in Bihar was grown within the state.

### Geographic extent of lead chromate turmeric adulteration across Bihar and neighboring states

**Fig. 1:**
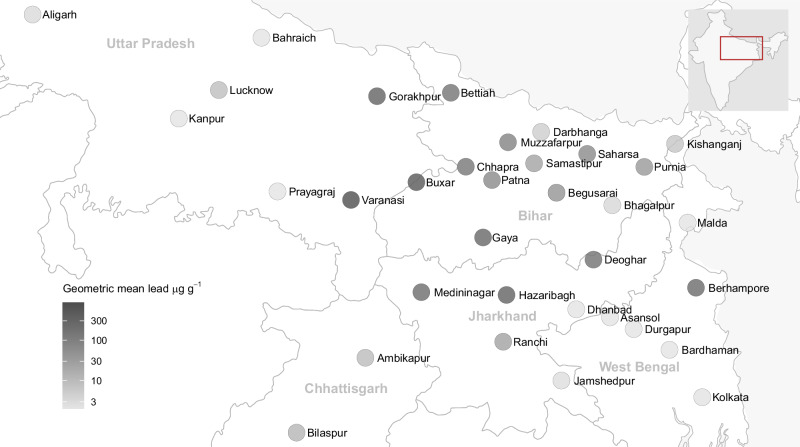
Geometric mean turmeric lead concentrations (µg g⁻¹) in each sampled city across Bihar, Jharkhand, Uttar Pradesh, West Bengal and Chhattisgarh 2021-2023 (n = 503).

**Fig. 2:**
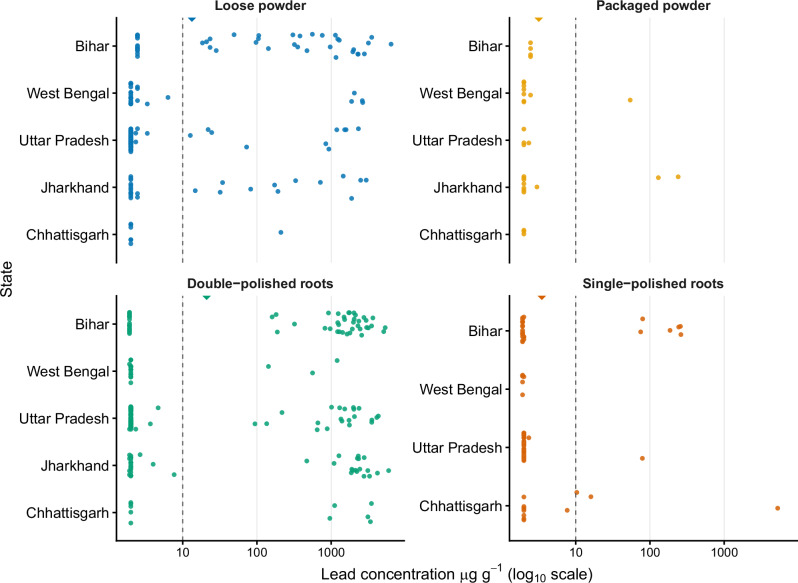
Turmeric lead levels by state of sale and turmeric type from the 2021–2023 systematic assessment across Bihar and neighboring states (n = 503).

A total of 503 turmeric root and powder samples and 80 chili and coriander samples were collected from 34 cities across five states in eastern India between 2021-2023 (Fig. 1, Fig. 2, Supplementary Tables 2 and 3). Samples were collected from a minimum of five cities in Bihar, Jharkhand, Uttar Pradesh and West Bengal along with two cities in Chhattisgarh. Only one sample of chili powder and four samples of coriander powder contained detectable lead, with a maximum of 573 µg g⁻¹. Overall, 30% of the turmeric samples (n = 153) across 25 cities had lead levels above 10 µg g⁻¹. Turmeric samples exceeded the threshold most often in Bihar followed by Jharkhand, Uttar Pradesh, and Chhattisgarh, with the least lead in samples from West Bengal (Fig. 1, Fig. 2). Every city in Bihar showed evidence of lead chromate turmeric adulteration, with a geometric mean of 48 (sd: 23) µg g⁻¹ and a maximum of 6,416 µg g⁻¹. Every sample of turmeric root and powder with above 10 µg g⁻¹ also contained chromium at levels suggestive of lead chromate^12^.

**Fig. 3:**
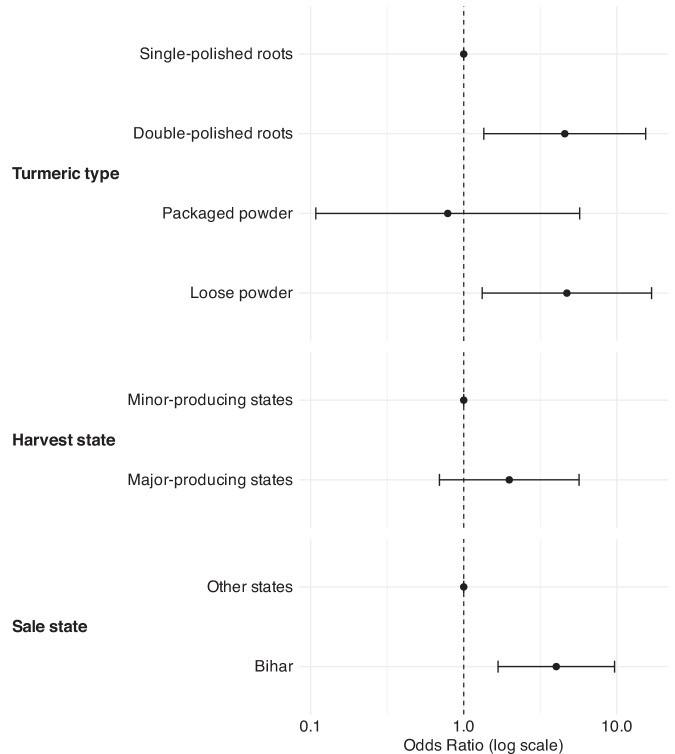
Odds ratios from a logistic regression accounting for clustering at the city level. The outcome variable is turmeric lead levels above 10 µg g⁻¹ and predictor variables are turmeric type, state of harvest, and state of sale (more regression details in Supplementary Table 4).

Logistic regression indicated the odds of exceeding 10 µg g⁻¹ lead were more than four times higher for double-polished roots and loose powder compared to single-polished roots (p = 0.004 and 0.007) and for turmeric sold in Bihar versus neighboring states (p = 0.001) (Fig. 3, Supplementary Table 4). Turmeric harvested in major versus minor turmeric producing states showed higher odds of lead over 10 µg g⁻¹ but the 95% confidence interval included 1 (p = 0.198). There was not a strong relationship with lead levels and price in Bihar in 2021 except at the lowest prices in the spline model (Supplementary Table 5).

### Practices and incentives for turmeric adulteration

The interviews indicated that spice adulteration with multiple chemicals was occurring at various points in the supply chain in harvesting regions and at facilities within or around local markets. Interviews and sample testing revealed that lead-free chemicals from hardware stores like alum (typically potassium aluminum phosphate) and iron oxide hydroxide, as well as “food-grade” colorants like tartrazine and oil yellow, were being added during boiling, polishing and grinding. Lead chromate, on the other hand, was reported to be principally added to turmeric roots as part of the polishing process, not during boiling or grinding. Turmeric roots typically get polished at harvesting regions in a large spinning drum; due to friction, the outer turmeric root skin rubs away and a layer of turmeric powder naturally forms, giving the roots a smooth appearance. Several interviewees described how polished turmeric roots sometimes undergo another round of mixing with lead chromate closer to the point of sale. After polished turmeric roots arrive at the market, vendors could add pigment by themselves to fix color discrepancies. When larger-scale coloring was needed, turmeric could also be sent to local processing facilities closer to the markets, although this was reported to be more costly.

Interviewees mentioned that traders requested lead chromate adulteration for two reasons: 1) to increase the bright appearance of turmeric roots and satisfy consumer demand, and 2) to fill in holes from insect damage and prevent further insect damage, extending the shelf-life of the roots. To that end, traders noted that lead chromate turmeric adulteration was more common late in the year when the roots have been stored for the longest since harvest (typically January-March). Generally, adding lead chromate was considered a tactic to sell lower-quality turmeric by creating a more attractive appearance. Although adulteration was illegal, respondents did not indicate that they were concerned about legal punishment.

Lead chromate pigment powder was available across many cities, sold in hardware stores in packets ranging from 100 grams to 25 kilograms and costing US$ 1.62 – 5.40 per kilogram (INR 120 – 400, using the January 1, 2022 exchange rate of US$ 1 = 74 INR). Traders selling lead chromate mentioned that it was primarily sold for staining wood and as a pigment in plastics and paint. They did not acknowledge the use of lead chromate in spices.

### Turmeric adulteration and patterns by source and grade

Interviews and sampling indicated that there were at least three grades of turmeric root and powder available in the market catering to different consumer segments. These grades were based on turmeric variety and root appearance: size, color, texture, smell, and evidence of insect damage. Turmeric root prices per kilogram were highest for Grade A roots that were large, smooth and intact, and lowest for Grade C roots that were broken, damaged or small. The 139 turmeric root samples from delivery trucks at the market hailed from major turmeric-producing locations including Erode (Tamil Nadu), Mysore (Karnataka), Sangli (Maharashtra) and Nizamabad (Telangana). The majority of samples were Grade B; and 62-90% of samples from each source state had elevated lead indicative of lead chromate adulteration (Supplementary Tables 6 and 7).

An additional 51 samples of turmeric were collected from the two major cities in Jharkhand and Uttar Pradesh with no previous evidence of lead chromate: Dhanbad and Prayagraj. None of these samples contained detectable lead even though they were predominantly Grade B and from the same source states as the lead-tainted turmeric sold in Bihar.

### Estimated blood lead levels (BLLs), IQ and economic impact

Based on the geometric mean powdered turmeric lead level in Bihar of 51 µg g⁻¹, we estimate eliminating lead chromate adulterated turmeric exposure could reasonably decrease child BLLs by 7.7-38.0 µg L⁻¹. Considering average BLLs among children in Bihar of 76 µg L⁻¹, we estimate a BLL shift to 68.3 to 38.0 µg L⁻¹ without turmeric lead exposure^21^. This translates to a ΔIQ of 0.3-2.3 points. Given approximately 18.6 million children 0-6 years old in Bihar according to the latest census (2011), the approximate size of a single birth cohort would be 2.7 million^22^. Therefore, we estimate an increase of 0.9-6 million IQ points per year, equivalent to US$ 239 million to US$ 1.6 billion. If cardiovascular disease mortality reductions are included, there would be an additional annual benefit of approximately US$ 430 million to US$ 2.8 billion. We used the powdered turmeric geomean because XRF analyses are most precise for powdered samples, and these results are consistent with estimates derived from the full sample geomean (within 6-12%).

### Regulatory influences on turmeric adulteration

Each state in India has its own State Food Authority, responsible for implementing and enforcing regulations in collaboration with the national-level Food Safety Standards Association of India (FSSAI). The State Food Authority consists of a Commissioner of Food Safety who oversees high-level policy and strategy across the state, and district-level Food Safety Officers who oversee the implementation of policy, licensing and sample testing.

Interviews with state- and district-level food safety government officials highlighted several themes. First, Food Safety Officers had numerous responsibilities across all aspects of food safety and often lacked manpower or resources for sample testing and enforcement. Responsibilities included oversight across thousands of businesses; from manufacturers, processors and vendors at street shops, bazaars and supermarkets, to dining establishments like street carts, restaurants and hotels. Interviewees mentioned that sweets, dairy, wheat, and oil were high risk commodities and priorities for inspection. Additionally, the overall human resource capacity of the State Food Authority varied by state and was not necessarily proportional to population.

Second, the top food safety priorities were generally those that were highly visible or caused acute symptoms. Unlike an *E. coli* outbreak, the adverse effects of lead exposure are typically invisible and take years to manifest. Food Safety Officers described how they responded to orders from the Commissioner as well as customer complaints; conducting a mix of routine scheduled inspections, inspections in response to customer complaints and raids. Some inspectors mentioned sample collection quotas, although this was not always the case and there was heterogeneity in sampling frequency and regularity. Customer complaints tended to focus on visible issues like hygiene violations at restaurants and the sale of expired or spoiled food. These were generally also easier for Food Safety Officers to identify as they did not always require lengthy lab testing.

Finally, for turmeric specifically, there was a general level of awareness about turmeric adulteration but it was not a prioritized issue; there was more emphasis on the adulteration of turmeric powder with food-safe ingredients and less focus on lead chromate adulterated turmeric roots. Within each state, Food Safety Officers were aware of lead chromate adulteration of turmeric, but awareness of lead chromate’s toxicity vis-à-vis other food safety concerns varied. Inspections focused on marketplaces and grinding mills but not turmeric root polishing facilities. The frequency of vendor-reported inspections for turmeric varied from monthly to not at all. Several Food Safety Officers mentioned that a critical barrier to policy enforcement was sample lab testing to confirm lead chromate adulteration, which was costly and took nearly two weeks to get results. Although respondents mentioned that they could tell when roots have yellow pigment just by looking at them, regulatory action required lab confirmation of lead chromate adulteration.

The penalty associated with food adulteration depended on the type and severity of the offense. Penalties were increasingly severe for repeat offenders, often starting with a warning or small fine, but escalating to larger fines, business closure and imprisonment. Food Safety Officers mentioned that fines could be up to US$ 1,350 (INR 100,000) and months of imprisonment. Officers in Bihar, Jharkhand and Uttar Pradesh were familiar with lead chromate turmeric adulteration violations in their states but had not been personally involved.

## Discussion

This study evaluated the geographic extent of lead chromate turmeric adulteration in eastern India. Consistent with recent evidence collected in 2020-2021, this study underscores the problem of widespread lead chromate turmeric adulteration across Bihar^17^. All 15 cities assessed in Bihar had evidence of lead chromate turmeric adulteration, with more than half of samples containing elevated lead exceeding the 10 µg g⁻¹ FSSAI limit. Additionally, this study provides the first evidence of lead chromate turmeric adulteration in other states neighboring Bihar. Turmeric lead levels exceeded the 10 µg g⁻¹ limit in at least one city in every state assessed, except West Bengal. Maximum turmeric lead concentrations were 5,000-6,000 µg g⁻¹, hundreds of times higher than the FSSAI limit. The results indicate that elevated turmeric lead levels are more likely in loose powder and polished turmeric roots than other forms of turmeric.

Eastern Indian states have some of the highest rates of turmeric consumption in the country^1^. This study suggests that lead chromate turmeric adulteration may be poisoning over 160 million people across the 34 sampled cities^23^. At the same time, Bihar and neighboring states suffer from the country’s highest rates of poverty and have poor health and development indicators. Thus, lead poisoning from turmeric may have a multiplicative adverse impact. For example, iron-deficiency anemia is a major public health problem in Bihar, impacting more than 60% of children, and anemic individuals absorb lead more readily^24,25^.

Although lead is toxic across all systems in the body, if we consider only the cognitive impacts from consuming turmeric with geometric mean lead levels observed in Bihar (48 µg g⁻¹), we estimate that eliminating lead chromate adulterated turmeric would increase IQ by 0.3-2.3 points per child and expected lifetime earnings by 1.4-4%^26^. These IQ gains in Bihar would be equivalent to up to US$ 1.6 billion in discounted lifetime earnings each year^10^. Past studies suggest the cost of addressing lead chromate adulteration is relatively low; less than US$ 1 million was spent to reduce lead chromate turmeric adulteration in Bangladesh with a slightly larger population than Bihar^16^. Therefore, eliminating lead chromate turmeric adulteration could have enormous returns on investment and boost the socioeconomic outlook for the state of Bihar, improving IQ and increasing earning potential.

While the conservative estimates of the impacts in Bihar and neighboring states are staggering, more people may be affected across India. Prior studies have provided evidence of lead-tainted turmeric originating from other states in India, including Assam, Tamil Nadu and Andhra Pradesh^17,27^. Interview respondents in the present study revealed that lead chromate was being added to turmeric roots locally by traders, but market sampling results also suggested that the predominantly middle grade turmeric arriving from the top five major producing states – which collectively produce more than two-thirds of the nation’s turmeric – was already adulterated with lead chromate. This suggests that the problem of lead chromate turmeric adulteration may not be restricted to the states that were the focus of this study.

Our evidence is consistent with past research, determining that lead chromate is added to turmeric roots to improve root appearance and color^6^. The present study also highlighted a new reason for adding lead chromate to roots; to prevent insect damage and enhance shelf-life. Notably, we did not find evidence of lead chromate being added to chili or coriander, unlike other studies^18,28^. Given that grinding equipment is shared between spices, periodic cross-contamination of chili, coriander or other non-turmeric-spices may be expected from lead chromate residue left on grinding equipment.

Having a food safety policy disallowing lead in spices is important but our study results indicate that it is insufficient to ensure a lead-free spice supply chain on its own. In other settings, lead chromate adulteration of spices was stopped through a combination of targeted awareness-raising, particularly with influential members of the government and the spice industry, as well as improved lead screening, and ultimately policy enforcement^16,29^. In Bangladesh, lead chromate had been added to turmeric for four decades and a policy was in place for several years without enforcement. The discovery that lead chromate adulterated turmeric was a source of lead poisoning catalyzed a nationwide intervention in 2019. Within a few months, there was a 90% reduction in the prevalence of lead-tainted turmeric, an improvement that was sustained for several years^16^. There is also evidence of reductions in spice and blood lead levels following similar intervention in the Republic of Georgia^29,30^.

Given the state-wise segregation of the Food Safety Authority in India and multiple points in the supply chain where lead chromate may be added to turmeric, both state- and national-level interventions are needed. Although regulation exists disallowing lead in turmeric in India, this is not routinely enforced. Necessary actions to overcome policy enforcement barriers include raising awareness and elevating lead chromate turmeric adulteration as a top tier food safety priority. Our interviews highlighted that Food Safety Officers and those in the turmeric industry did not fully understand the importance of lead chromate’s toxicity relative to other issues. Moreover, since lead’s adverse effects are silent and long-term^31^, the issue does not fall into the urgent and acute category, which remain the focus of customer complaints and the department’s priority.

Additional barriers to overcome include the limited capacity for sample collection and delays incurred by lab testing, including multi-step methods such as inductively coupled plasma mass spectrometry (ICP-MS) and colorimetry. Turmeric roots are widely traded across the region, and our interviews suggested that it may be possible for an informed individual to identify highly adulterated turmeric roots just by looking at them, but the efficacy of this approach is unknown. Moreover, given that it is not possible to determine if turmeric powder has elevated lead levels by visual inspection, improved rapid lead screening with portable x-ray fluorescence analyzers could support more immediate actions and consistent policy enforcement across the region. Although x-ray fluorescence analyzers cost tens of thousands of dollars, this is very little compared to the economic burden of lead chromate turmeric adulteration.

This study has two primary limitations. First, the phased cross-sectional sampling during the summer season may underestimate the extent of lead chromate turmeric adulteration in other seasons. Given that lead chromate was reported to be added to roots to enhance appearance and cover up insect damage from long storage, it is likely that the prevalence of lead-tainted roots would be lower in the spring when the recent harvest is fresh, and higher in the winter when roots have been stored for months. The data from this study provide a general estimate of the magnitude of the problem during summer, but subsequent repeated sampling efforts would allow for more nuanced interpretations and comparisons, especially in different seasons and between states. Second, the field data collectors did not directly observe the process of lead chromate turmeric adulteration; evidence about this comes entirely from interviews and sampling. Given that lead chromate turmeric adulteration is illegal, some respondents were not forthcoming about personal involvement in spice adulteration and spoke about it in general terms. Therefore, some details remain unclear related to where and how often lead chromate turmeric adulteration occurs.

Lead exposure affects hundreds of millions of people and causes trillions of dollars in lost productivity in low- and middle-income countries per year. This study highlights the magnitude of lead chromate turmeric adulteration in five eastern states of India, impacting over 160 million people daily. Importantly, it highlights where lead chromate enters the turmeric supply chain, as well as the incentives driving the practice. Since data collection took place during the summer season, closest to turmeric season, this study may underestimate exposure later in the year. Given the overwhelmingly elevated lead levels in turmeric in India, immediate follow-up efforts to enforce food safety policy and increase awareness among industry actors and consumers are needed.

## Methods

### Systematic turmeric sampling across Bihar and neighboring states to understand the geographic extent of adulteration

The study team sampled turmeric from a total of 34 cities across the region during the summer season in 2021 and 2023. First, the study team sampled turmeric from 13 of the largest cities across Bihar with populations above 100,000 people as well as another 8 cities in bordering states. Second, turmeric was sampled from 13 of the nearest similarly-sized cities in eastern Uttar Pradesh, northern Jharkhand, western West Bengal, and Chhattisgarh (Fig. 1).

The largest wholesale and retail spice markets per city were visited following a similar protocol as described in Forsyth et al. 2024^17^. The team identified the different varieties of turmeric for sale and anonymously purchased every unique type of turmeric available. Turmeric types varied based on the physical form (e.g., dried root and loose or packaged powder), the degree of processing (e.g., single-polished vs. double-polished roots), and harvesting location. One sample of each unique type of turmeric was purchased and stored in double-layered polyethylene bags to minimize contamination.

When available, samples of whole and ground chili and coriander were also sampled from each market following the same selection and storage practice.

### Mixed methods approach to assess patterns of lead chromate adulteration and regulatory influences: qualitative interviews and purposive sampling

Due to the evidence of lead chromate adulteration in Bihar, a subsequent mixed-methods sampling-based and qualitative investigation was conducted across the state in August-December 2023. Local field research partners trained in value chain investigation mapped the turmeric supply chain, beginning in the Samastipur district where the majority of Bihari turmeric is grown. The field researchers identified major trading hubs: cities where large volumes of turmeric are sold and distributed to other cities across the state. Based on the findings of the 2021-2023 systematic turmeric assessment and the supply chain investigation in 2023, five trading hubs were selected for further sampling and qualitative study. These included the three major trading hubs in Bihar with a high prevalence of lead chromate adulteration (Patna, Gaya and Bhagalpur), as well as one hub each in Uttar Pradesh (Prayagraj) and Jharkhand (Dhanbad), both without evidence of lead chromate adulteration.

As feasible, additional samples of turmeric were collected from these trading hubs to assess patterns of lead chromate addition by grade and source in December 2023. Every available turmeric type across each grade was sampled. Samples included turmeric for sale at the wholesale markets, as well as turmeric from burlap sacks collected from delivery trucks arriving at the market. The grade, source state, price, and lead and chromium concentrations were recorded and analyzed.

Additionally, further qualitative investigation explored the incentives driving lead chromate adulteration and the regulatory context in December 2023-2024. Trained social scientists conducted semi-structured interviews and observations with turmeric farmers, traders, millers, wholesalers, retailers and food safety officers in Bihar, Uttar Pradesh and Jharkhand. Core focal areas of inquiry included: value chain structure, incentives and reasons for turmeric adulteration (by grade, source, location), food safety policy enforcement, and regulatory priorities. Samples of chemicals used in turmeric processing were also collected and analyzed. The field researchers obtained informed consent from study participants. The ethical review committees at Sigma in India (#10039/IRB/21-22) and Stanford University in the United States (#62424) reviewed and approved the protocol.

### Sample analysis

Samples were analyzed following the approach outlined in Lopez et al.^28^. Samples of 20 g of turmeric powder or roots were placed in lead-free, uniformly sized (7.5 ×10 cm) polyethylene bags (~25 µm thick) which resulted in a sample layer of at least 2 cm, ensuring sufficient thickness for analysis via energy-dispersive x-ray fluorescence spectrometry. Sample bags were wiped free of dust prior to analysis. Because turmeric powder is ground to a uniform powder consistency for sale at the market, no extra preparation was needed. For roots, the thickest root was used for measurement and replicate measurements were taken from different parts of the sample. Lead concentrations were measured in triplicate using a handheld Olympus DELTA Classic Plus (DCC-4000) X-ray fluorescence analyzer equipped with a Ta anode X-ray tube excitation source (4 W, 200 mA maximum) and a silicon-PIN detector. Lead was quantified using Lβ (12.6 keV) X-ray peaks in Soil Mode with typical measurement times of 60 seconds. Counts were normalized to the Compton and Rayleigh scattering peaks to correct for matrix effects. The effective limit of detection was 4 µg g⁻¹ for lead determined by the Olympus software which used spectral variance and the 3-sigma rule to separate signal from noise by defining the minimum detectable concentration as three times the standard deviation of background noise.

Empirical calibration curves were built from a subset of samples of turmeric powder and root samples prepared exactly as the samples for analysis with XRF but also analyzed via inductively coupled plasma mass spectrometry (ICP-MS, Thermo Scientific XSERIES 2) at Stanford’s Environmental Measurements Facility (em1.stanford.edu). Prior to ICP-MS analysis, powder samples were sub-sampled (0.5 grams) and digested in 69% trace-metal grade nitric acid (MarsXpress, CEM). Dried turmeric roots were also sub-sampled (2–7 g) and soaked in 35% trace-metal grade nitric acid for 30 minutes after which the solution was further microwave digested. Samples were further diluted to 2% nitric acid for analysis via ICP-MS alongside internal standard solution and blanks for quality control. We developed separate calibration curves for powders and roots due their differing measurement characteristics. XRF analysis provides quantitative measurements of lead in turmeric powders within 5%, as they are ground to uniform particle sizes and exhibit homogeneous texture^28^. In contrast, turmeric roots are solid and more heterogeneous in texture, so while XRF analysis provides reliable semi-quantitative measurements within 24%, there is a tendency to slightly overestimate lead at higher concentrations^28^. Therefore, separate calibration curves allowed us to more accurately model lead levels for each turmeric type. In total, 7 turmeric powder samples and 9 turmeric root samples representing a range in lead concentrations from 4 – 6,094 µg g⁻¹ were used. Consistent with prior studies, XRF measurements showed excellent agreement with ICP-MS for powder samples (R^2^ = 0.99, mean percent error=0.82%) and good agreement for turmeric roots (R^2^ = 0.9798, mean percent error=18.36%)^28^ (Supplementary Figs. 3 and 4).

Descriptive statistics were calculated to understand patterns in turmeric lead levels. A logistic regression model included predictors of turmeric lead levels above 10 µg g⁻¹: turmeric type, state of sale and state of harvest for each sample. To account for intra-city sample correlation, we used Huber–White robust standard errors clustered at the city level. Due to the small number of cities in some states, we aggregated state of sale to two categories: within Bihar and outside of Bihar. Similarly, we aggregated state of harvest to two categories: major turmeric producing states, and minor turmeric producing states (conclusions were consistent with disaggregated models). The major turmeric-producing states were defined as Maharashtra, Telangana, Tamil Nadu, and Mahya Pradesh, and Karnataka that are consistently reported as being in the top five major producers^20^. Year of sampling was not added to the model because cities in Bihar were exclusively sampled in the first year. Although there is no evidence of interventions targeting turmeric lead chromate adulteration in India between 2021-2023 that could have altered lead levels, we cannot assess this.

Due to the two-staged sampling across a wide geographic area in two years, the relationship between turmeric lead levels and price was only examined among samples from Bihar during a single summer season in 2021. Due to the fewer number of samples in Bihar, this was an exploratory analysis focused on a reduced number of covariates. We compared separate models (logistic and gamma log link) with turmeric lead level as the outcome and turmeric type and price as predictors. Price was modeled as a i) continuous variable, and ii) spline function with two and three segments. Model fit was evaluated using the Akaike Information Criterion (AIC) and the Bayesian Information Criterion (BIC).

### Estimating BLL, IQ and economic losses associated with turmeric lead exposure

We estimated the annual cost of lead chromate adulterated turmeric exposure in Bihar in terms of lost future earnings for a single birth cohort. We calculated the increase in children’s blood lead levels (ΔBLL) that would be expected from consistently consuming turmeric with average lead levels in Bihar (geometric mean of turmeric powder: 51 µg g⁻¹), compared to turmeric below 10 µg g⁻¹ using three approaches. The first approach used basic assumptions per the following formula as described in in Forsyth et al.^17^:

$$\Delta {BLL}={TurmCons}\left(\frac{g}{{day}}\right)x\,{TurmPb}\left(\frac{\mathrm{\mu g}}{{\rm{g}}}\right)x\,0.429\,x\frac{0.52\,\left(\frac{\mathrm{\mu g}}{{\rm{L}}}\right)}{1\left(\frac{\mathrm{\mu g}}{\mathrm{day}}\right)}$$

where*:*

Δ*BLL* is the change in child blood lead level.

*TurmCons* is the estimated grams of turmeric consumed per child per day, ~0.7 in Bihar.

*TurmPb* is the turmeric lead concentration, ~51 in Bihar.

0.429 is average bioaccessibility of lead in turmeric

0.52 μg L⁻¹ is the estimated increase in BLL per μg of lead ingested per day

The second and third approaches relied on the same assumptions for turmeric consumption, turmeric lead levels and bioaccessibility, but estimated BLLs via models developed by the U.S. Environmental Protection Agency: the All Ages Lead Model (AALM) version 3.0 for adults and children of any age and the Integrated Exposure Uptake Biokinetic (IEUBK) Model for young children. For both models, we set all other lead consumption to zero and modeled daily ingestion of the bioaccessible portion of lead in turmeric between 2 and 3 years old.

We assumed that children’s BLLs (considering all sources of exposure) would be equivalent to average values measured in a recent state-wide study by Lu et al.^21^. Without turmeric lead exposure, we assumed BLLs would be reduced by the amount attributable to turmeric as calculated above (ΔBLL). To estimate the associated IQ loss (ΔIQ), we used the non-linear relationship based on Lanphear et al.^32^: ΔIQ = (-3.25) x [(ln(BLL) –ln(BLL_0_)]. This provides a conservative estimate given the steeper IQ shift at lower BLLs.

We estimated present value of lifetime earnings equivalent to US$ 12,940 using the following formula and assuming working ages 15-60, average real annual earnings of US$ 1,359/year in Bihar, current age of 3 years old, a workforce participation rate of 53.2% and a discount rate of 3%^33^.

$${PV}=\rho \mathop{\sum }\limits_{t=18}^{60}\frac{{E}_{t}}{{(1+d)}^{t-{t}_{0}}}$$

where:

E_t_=annual salary, US$ 1,359/year

d=discount rate, 0.03

ρ=workforce participation rate, 0.532

t_0_=age now, 3 years old

We assumed that each IQ point results in 2% higher earnings^34^; so we multiplied 2% by ΔIQ by the present value of lifetime earnings to get increased lifetime earnings without turmeric lead chromate adulteration. We calculated lower and upper bounds for gained lifetime earnings based on the estimates for ΔBLL and multiplied by the size of the birth cohort. We estimate the additional benefit from cardiovascular disease mortality reductions using the Larsen and Sánchez-Triana (2023) finding that long-term cardiovascular disease mortality costs were 1.8 times those associated with IQ loss in India in 2019^10^.

## Supplementary information

Supplementary information

## Data Availability

The data that support the findings of this study are available in Supplementary Table 8.
