# Supplementary material for: Systemic drivers of toxic food adulteration: lead chromate in turmeric across eastern India
Source: NPJ Sci Food. 2026 May 6;10:220. doi: 10.1038/s41538-026-00867-8 (PMC13357593; doi:10.1038/s41538-026-00867-8)
Supplement: Supplementary file 1 — Supplementary information [file 41538_2026_867_MOESM1_ESM.pdf]

Systemic drivers of toxic food adulteration: lead chromate in turmeric across eastern India

Jenna E. Forsyth[1,2], Manu Sinha[3], Amogh Bandekar[2], Dinsha Mistree[4], Manoj Parida[5], Emily Nash[6], Lavanya Nambiar[6], Christlee Elmera[2], Stephen P. Luby[1,2]

[1]School of Medicine, Stanford University, Stanford, California, U.S.A.

[2]King Center on Global Development, Stanford, California, U.S.A.

[3]Frameworks, Bhopal, India

[4]Hoover Institution, Stanford University, Stanford, California, U.S.A.

[5]DCOR, New Delhi, India

[6]Pure Earth, New York City, U.S.A.

Corresponding author: Jenna E. Forsyth, Stanford School of Medicine, 473 Via Ortega, Y2E2 Building, Suite 226, Stanford, CA 94305. Phone: 435-232-2955, email: jforsyth@stanford.edu

Running title: Adulteration of turmeric with lead chromate in India

Table of Contents

Figure S1. Turmeric value chain map. 3

Figure S2. Supply chain of turmeric from within and outside in the state of Bihar. 4

Figure S3. Left) Calibration curve for 7 turmeric powder samples with lead concentrations ranging from 8 to 4,537 µg g-1 as measured by ICP-MS. 6

Figure S4. Left) Calibration curve for 9 turmeric root samples with lead concentrations ranging from 4 to 6,094 µg g-1 as measured by ICP-MS 7

Table S1. Summary of stakeholders who participated in qualitative interviews. 8

Table S2. Summary of turmeric lead concentrations of polished roots and loose powder from systematic assessments in Bihar and neighboring states, 2021-2023 (n=503). 9

Table S3. Summary of lead concentration and price of all samples of single- and double-polished roots, and loose and packaged turmeric powder from the 2021-2023 systematic assessment across Bihar and neighboring states (n=503). 10

Table S4. Odds ratios from a logistic regression accounting for clustering at the city level with turmeric lead levels above 10 µg/g as the outcome variable and turmeric type, state of harvest, and state of sale as predictors. 11

Table S5. Gamma log link regression of samples from Bihar in 2021 with turmeric lead level as the outcome and turmeric type and price, modeled as a piecewise linear spline, as predictors 12

Table S6. Source and grade-wise lead chromate adulteration of 138 turmeric roots sampled off delivery trucks before being displayed by traders (Patna, Gaya, and Bhagalpur, Bihar – 2023). 13

Table S7. Grade-wise turmeric lead chromate adulteration of turmeric roots and powder from the major markets in trading hubs in Bihar, 2023 (n=98; source states included Tamil Nadu, Telangana, Karnataka, Maharashtra, Odisha and Bihar). 14

Table S8. Turmeric market sample data used in analyses (n=503). 15

Turmeric wholesale/vendor/businessperson interview guide 29

Turmeric grinder interview guide 33

Turmeric polisher interview guide 34

Turmeric farmer interview guide 36

Food safety authority/inspector/police interview guide 38

Yellow pigment wholesaler/vendor/businessperson interview guide 39

Description of qualitative methods 40

Figure S1. Turmeric value chain map.

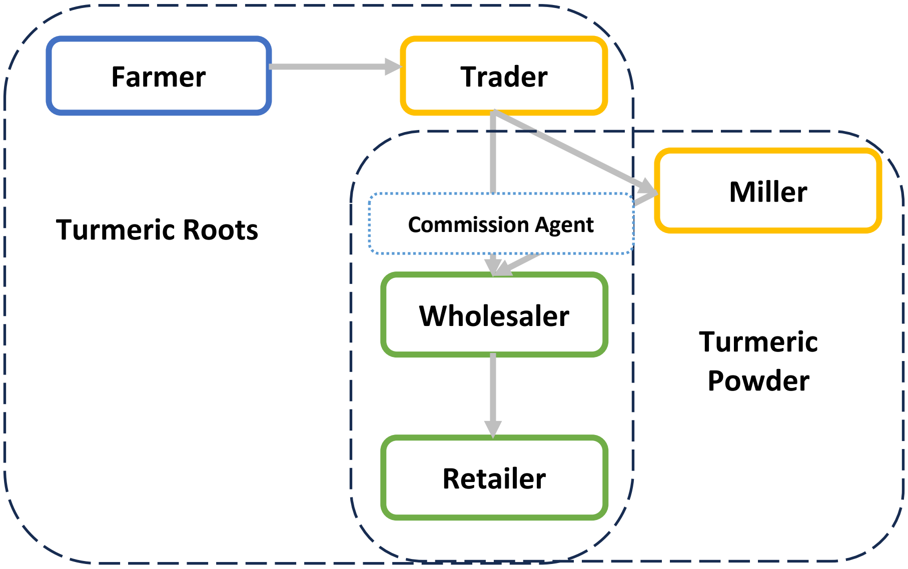

Figure S2. Supply chain of turmeric from within and outside in the state of Bihar.

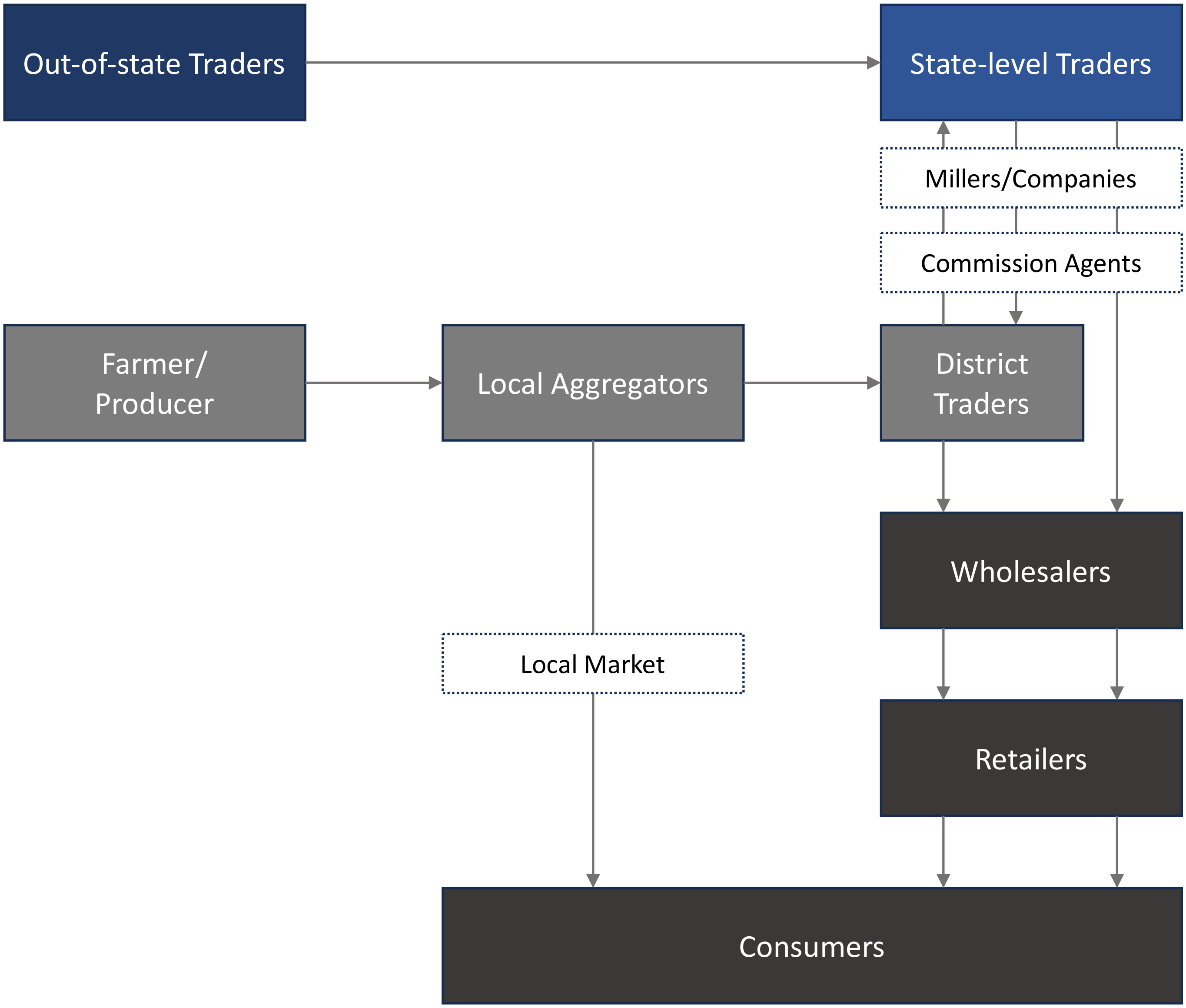

88

Figure S3. Left) Calibration curve for 7 turmeric powder samples with lead concentrations ranging from 8 to 4,537 µg g-1 as measured by ICP-MS. Right) Calibration curve for 3 low-range powder samples ranging from 8 to 223 µg g^{-1}. Standard error bars displayed. Mean relative standard deviation was 3.96% for XRF and 2.5% for ICP-MS.

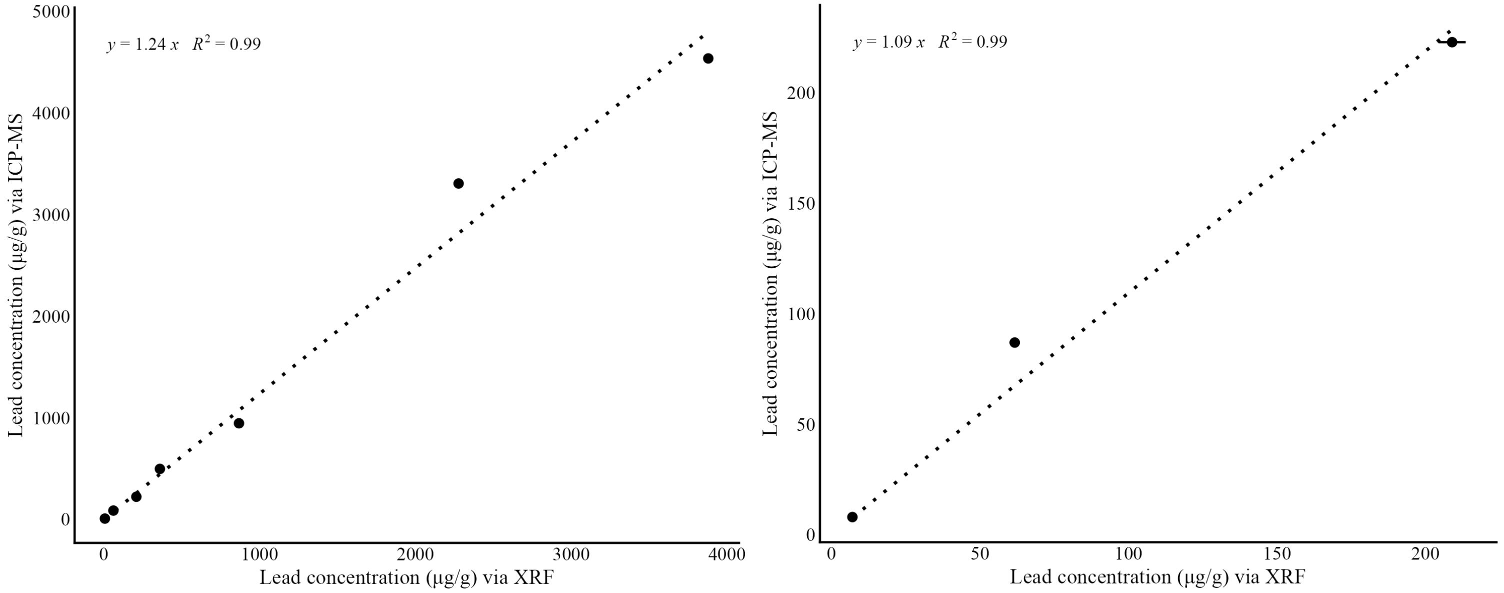

Figure S4. Left) Calibration curve for 9 turmeric root samples with lead concentrations ranging from 4 to 6,094 μg g-1 as measured by ICP-MS. Right) Calibration curve for 4 turmeric root samples with lead concentrations ranging from 4 to 146 μg g^{-1}. Standard error bars displayed. Mean relative standard deviation was 2.48% for XRF and 2.5% for ICP-MS.

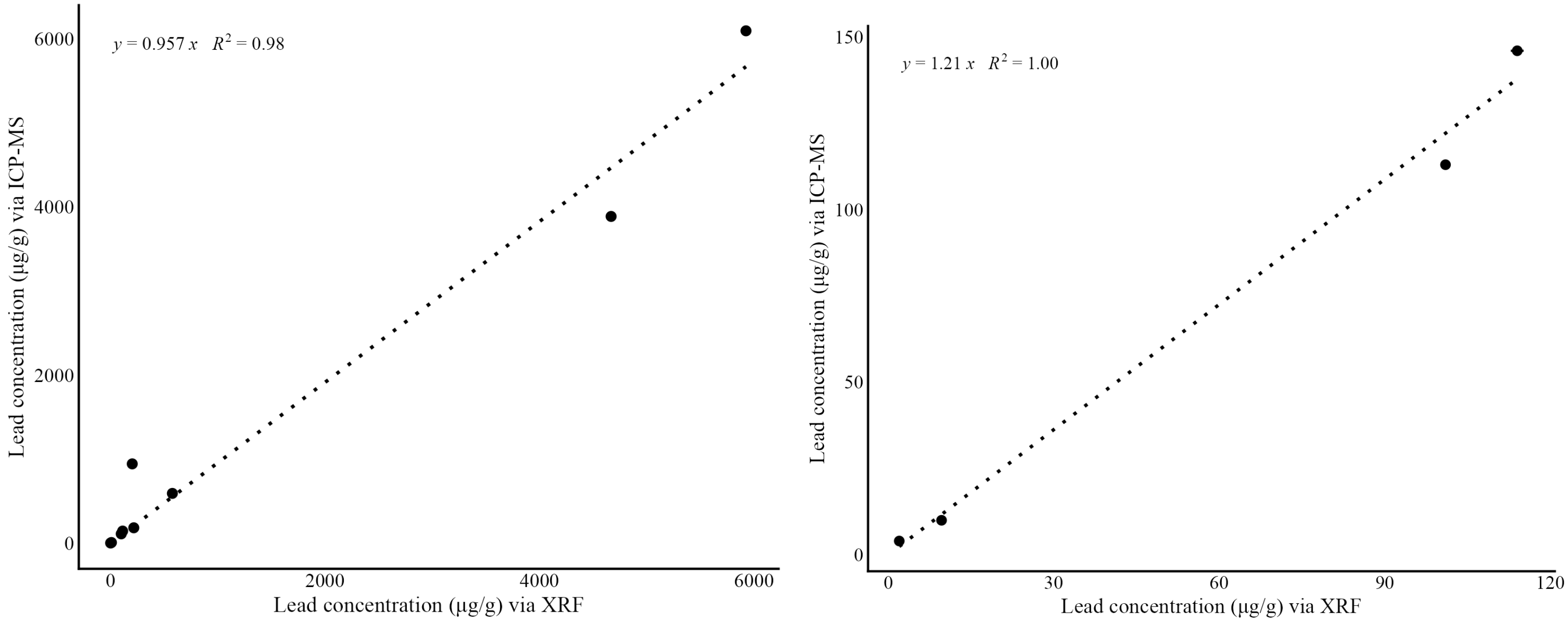

Table S1. Summary of stakeholders who participated in qualitative interviews.

Value Chain Role	Number of Interviews
Farmers	5
Spice Traders	61
Spice Millers	11
Spice Wholesalers	8
Spice Customers	10
Others[1]	3
Regulatory (Food safety inspectors and law enforcement officers)	27
Pigment Traders	3

[1] Commission agent, retailer, government research groups

Table S2. Summary of turmeric lead concentrations of polished roots and loose powder from systematic assessments in Bihar and neighboring states, 2021-2023 (n=503).

State	n	Median	90th Percentile	Maximum	n (%) over 10
Bihar	134	39	2456	6416	72 (54%)
Chhattisgarh	32	<LOD	3125	5255	9 (28%)
Jharkhand	77	<LOD	2497	5950	32 (42%)
Uttar Pradesh	200	<LOD	908	4320	32 (16%)
West Bengal	60	<LOD	352	2662	8 (13%)
	**503**	**<LOD**	**2011**	**6416**	**153 (30%)**

Table S3. Summary of lead concentration and price of all samples of single- and double-polished roots, and loose and packaged turmeric powder from the 2021-2023 systematic assessment across Bihar and neighboring states (n=503).

**Turmeric Type**	**n**	**Lead Concentration µg g⁻¹**			**Price per KG (₹)**
		**n (%) <10**	**n (%) 10-100**	**n (%) >100**	**Mean (SD)**
Loose powder	162	105 (65)	15 (10)	42 (26)	229 (49)
Packaged powder (no brand)	19	16 (85)	1 (6)	2 (11)	240 (56)
Packaged branded powder	11	11 (100)	0 (0)	0 (0)	260 (60)
Double-polished roots	235	152 (65)	1 (1)	82 (35)	207 (57)
Single-polished roots	76	66 (87)	5 (7)	5 (7)	196 (58)
**TOTAL**	**503**	**350 (70)**	**22 (5)**	**131 (27)**	**215 (56)**

Table S4. Odds ratios from a logistic regression accounting for clustering at the city level with turmeric lead levels above 10 µg/g as the outcome variable and turmeric type, state of harvest, and state of sale as predictors.

Variable	Odds Ratio	95% CI, lower	95% CI, upper	P-value	Significance
**Turmeric Type**					
Single-polished roots	reference				
Double-polished roots	4.57	1.36	15.32	0.014	**
Loose powder	4.72	1.33	16.73	0.016	**
Packaged powder	0.79	0.11	5.65	0.811	
**State of Harvest**					
Minor turmeric-producing states[1]	reference				
Major turmeric-producing states[2]	1.98	0.70	5.63	0.198	
**State of Sale**					
Other states[3]	reference				
Bihar	4.02	1.68	9.60	0.002	***

* p-value <0.1, ** p-value <0.05, *** p-value<0.01

[1]Bihar, Uttar Pradesh, Jharkhand, West Bengal, Kerala

[2]Maharashtra, Tamil Nadu, Telangana, Madhya Pradesh

[3]Uttar Pradesh, Jharkhand, West Bengal, and Chhattisgarh

n=408, Likelihood ratio chi^2: $p = 0.0026$

Table S5. Gamma log link regression of samples from Bihar in 2021 with turmeric lead level as the outcome and turmeric type and price, modeled as a piecewise linear spline, as predictors.

Variable	Coefficient	95% CI, lower	95% CI, upper	P-value	Significance
**Turmeric Type**					
Single-polished roots	reference				
Doubl-polished roots	3.07	1.76	4.38	<0.001	***
Loose powder	-2.48	-3.82	-1.15	<0.001	***
Packaged powder	2.63	1.18	4.08	<0.001	***
**State of Harvest**					
Minor turmeric-producing states	reference				
Major turmeric-producing states	0.7431431	0.2217887	1.264497	0.005	***
**Price**					
Lowest tertile spline	0.01	-0.02	0.03	0.666	
Middle tertile spline	0.01	-0.03	0.05	0.653	
Highest tertile spline	-0.04	-0.09	0.02	0.168	

* p-value <0.1, ** p-value <0.05, *** p-value<0.01
n=116, Gamma log link regression accounting for city level clustering
Minor-producing States: Bihar, Jharkhand, Uttar Pradesh, West Bengal, Kerala

Table S6. Source and grade-wise lead chromate adulteration of 138 turmeric roots sampled off delivery trucks before being displayed by traders (Patna, Gaya, and Bhagalpur, Bihar – 2023).

**Source**	**Samples exceeding 10 µg/g Pb threshold n (%)**	**Grade**	**Grade-wise samples exceeding 10 µg/g Pb threshold n (%)**	**Total samples (n)**
Erode (Tamil Nadu)	42 (81)	A	8 (89)	9
		B	35 (81)	43
		C	0 (0)	1
Mysore (Karnataka)	8 (62)	A	0 (0)	0
		B	8 (73)	11
		C	0 (0)	2
Sangli (Maharashtra)	35 (67)	A	9 (64)	14
		B	26 (72)	36
		C	0 (0)	2
Nizamabad (Telangana)	18 (90)	A	1 (100)	1
		B	17 (89)	19
		C	0 (0)	0
**TOTAL**	**104 (75)**	**A**	**18 (75)**	**24**
		**B**	**86 (75)**	**109**
		**C**	**0 (0)**	**5**

Table S7. Grade-wise turmeric lead chromate adulteration of turmeric roots and powder from the major markets in trading hubs in Bihar, 2023 (n=98; source states included Tamil Nadu, Telangana, Karnataka, Maharashtra, Odisha and Bihar).

Grade	Samples exceeding 10 $\mu g\ g^{-1}$ Pb threshold n (%)	Type	Samples exceeding 10 $\mu g\ g^{-1}$ Pb threshold n (%)	Total sample (n)
A	7 (47)	Root	6 (43)	14
		Powder	1 (100)	1
B	36 (84)	Root	35 (88)	40
		Powder	1 (33)	3
C	1 (10)	Root	1 (20)	5
		Powder	0 (0)	5
Unspecified	9 (45)	Root	6 (43)	14
		Powder	3 (19)	16
**TOTAL**	**53 (54)**	**Root**	**48 (66)**	**73**
		**Powder**	**5 (20)**	**25**

Table S8. Turmeric market sample data used in analyses (n=503). All_pb_avg: average sample lead level in $\mu g\ g^{-1}$. City_final: city of collection. Type_groupfull4: loose powder=1, packaged powder=2, double-polished roots=4, single-polished roots=5. Sellstate_bin: Bihar=1, Other=0. Turm_major: 1=major turmeric-producing states (Maharashtra, Tamil Nadu, Telangana, and Madhya Pradesh and Karnataka), 0=minor turmeric-producing states (Bihar, Uttar Pradesh, Jharkhand, Chhattisgarh, West Bengal, Kerala, and Andhra Pradesh).

sampid_	city_final	all_pb_avg	type_groupfull4	sellstate_bin	turm_major
15_BS_5_1_1	Dhanbad	2	1	0	1
15_BS_5_2_4	Dhanbad	3	3	0	1
15_BS_6_1_4	Dhanbad	2	3	0	1
15_BS_6_2_1	Dhanbad	2	1	0	1
15_BS_6_3_3	Dhanbad	2	2	0	0
15_BS_7_1_1	Dhanbad	2	1	0	1
15_BS_7_2_4	Dhanbad	2	3	0	1
15_PB_1_1_1	Dhanbad	2	1	0	
15_PB_1_2_4	Dhanbad	2	3	0	1
15_PB_2_1_1	Dhanbad	2	1	0	1
15_SM_3_1_2	Dhanbad	2	2	0	
15_SM_4_1_4	Dhanbad	2	3	0	
15_SM_4_2_1	Dhanbad	2	1	0	
18_PG_10_1_1	Lucknow	1521	1	0	0
18_PG_10_2_4	Lucknow	2	3	0	0
18_PG_10_3_6	Lucknow	1301	3	0	0
18_PG_12_1_1	Lucknow	2	1	0	0
18_PG_12_2_4	Lucknow	2	3	0	0
18_PG_12_3_6	Lucknow	1741	3	0	
18_RG_1_1_4	Lucknow	2	3	0	1
18_RG_1_2_1	Lucknow	2	1	0	1
18_RG_1_3_5	Lucknow	2	4	0	1
18_RG_1_4_6	Lucknow	2	3	0	1
18_RG_1_5_6	Lucknow	2	3	0	1
18_RG_2_1_1	Lucknow	2	1	0	0
18_RG_2_1_1	Lucknow	2	1	0	0
18_RG_2_2_4	Lucknow	2	3	0	0
18_RG_2_2_4	Lucknow	2	3	0	1
18_RG_2_3_5	Lucknow	2	4	0	0
18_RG_2_3_5	Lucknow	2	4	0	1
18_RG_2_4_6	Lucknow	2	3	0	1
18_RG_2_4_6	Lucknow	2	3	0	0
18_RG_2_5_6	Lucknow	2	3	0	1
18_RG_3_1_1	Lucknow	2	1	0	0

18_RG_3_2_4	Lucknow	2	3	0	1
18_RG_3_3_6	Lucknow	2	3	0	
18_RG_3_4_6	Lucknow	2	3	0	1
18_RG_3_5_5	Lucknow	2	4	0	
18_RG_4_1_1	Lucknow	2	1	0	0
18_RG_4_2_4	Lucknow	2	3	0	1
18_RG_4_3_5	Lucknow	2	4	0	1
18_RG_4_4_6	Lucknow	2	3	0	1
18_RG_4_5_6	Lucknow	2	3	0	1
18_RG_5_1_1	Lucknow	2	1	0	0
18_RG_5_2_4	Lucknow	2	3	0	0
18_RG_5_3_5	Lucknow	79	4	0	1
18_RG_5_4_6	Lucknow	1398	3	0	1
18_RG_5_5_6	Lucknow	2	3	0	1
18_RG_6_1_1	Lucknow	2	1	0	0
18_RG_6_2_4	Lucknow	2	3	0	1
18_RG_6_3_6	Lucknow	1012	3	0	1
18_RG_6_4_6	Lucknow	5	3	0	1
18_RG_7_1_1	Lucknow	2	1	0	0
18_RG_7_2_4	Lucknow	2	3	0	1
18_RG_7_3_6	Lucknow	663	3	0	1
18_RG_7_4_6	Lucknow	1997	3	0	1
18_RG_8_1_1	Lucknow	2	1	0	0
18_RG_8_2_4	Lucknow	218	3	0	1
18_RG_8_3_6	Lucknow	2376	3	0	1
18_RG_8_4_6	Lucknow	884	3	0	
18_RG_9_1_1	Lucknow	2	1	0	0
18_RG_9_2_4	Lucknow	2	3	0	1
18_RG_9_3_6	Lucknow	2	3	0	1
18_RG_9_4_6	Lucknow	648	3	0	1
26_GM_1_1_1	Muzzafarpur	2288	1	1	
26_GM_1_2_4	Muzzafarpur	1287	3	1	
26_GM_1_3_5	Muzzafarpur	2	4	1	
26_GM_1_4_7	Muzzafarpur	2	4	1	
26_GM_2_1_1	Muzzafarpur	354	1	1	0
26_GM_2_2_4	Muzzafarpur	2296	3	1	0
26_GM_2_3_5	Muzzafarpur	2	4	1	0
26_GM_2_4_6	Muzzafarpur	2	3	1	0
26_MM_3_1_1	Muzzafarpur	97	1	1	
26_MM_3_2_4	Muzzafarpur	3168	3	1	

26_MM_3_3_5	Muzzafarpur	75	4	1	
26_MM_4_1_1	Muzzafarpur	18	1	1	
26_MM_4_2_4	Muzzafarpur	1263	3	1	
27_GB_1_1_1	Darbhanga	2	1	1	0
27_GB_1_2_4	Darbhanga	2	3	1	0
27_GB_1_3_6	Darbhanga	2	3	1	0
27_GB_2_1_1	Darbhanga	21	1	1	0
27_GB_2_2_4	Darbhanga	2	3	1	0
27_GB_2_3_5	Darbhanga	261	4	1	0
27_GB_2_4_7	Darbhanga	2	4	1	0
27_TR_3_1_1	Darbhanga	2	1	1	
27_TR_3_2_4	Darbhanga	2	3	1	
27_TR_3_3_6	Darbhanga	2	3	1	
27_TR_4_1_1	Darbhanga	143	1	1	
27_TR_4_2_4	Darbhanga	2	3	1	
27_TR_4_3_5	Darbhanga	2	4	1	
28_GC_1_1_1	Kishanganj	472	1	1	0
28_GC_1_2_2	Kishanganj	2	2	1	0
28_GC_1_3_5	Kishanganj	2	4	1	0
28_GC_2_1_1	Kishanganj	2	1	1	0
28_GC_2_2_5	Kishanganj	2	4	1	0
29_BS_3_1_1	Purnia	106	1	1	1
29_BS_3_2_4	Purnia	188	3	1	1
29_BS_4_1_1	Purnia	49	1	1	0
29_BS_4_2_4	Purnia	2	3	1	0
29_BS_4_3_5	Purnia	80	4	1	0
29_KB_1_1_1	Purnia	28	1	1	0
29_KB_1_2_4	Purnia	181	3	1	0
29_KB_1_3_5	Purnia	2	4	1	0
29_KB_2_1_1	Purnia	1141	1	1	0
29_KB_2_2_4	Purnia	2	3	1	0
30_SC_3_1_1	Saharsa	1287	1	1	1
30_SC_3_3_5	Saharsa	2	4	1	0
30_SC_4_1_1	Saharsa	380	1	1	0
30_SC_4_2_4	Saharsa	921	3	1	1
30_SC_4_3_5	Saharsa	2	4	1	0
30_SM_1_1_1	Saharsa	2	1	1	0
30_SM_1_2_4	Saharsa	1995	3	1	0
30_SM_1_3_5	Saharsa	2	4	1	0
30_SM_2_1_1	Saharsa	2011	1	1	0

30_SM_2_2_4	Saharsa	2	3	1	0
30_SM_2_3_4	Saharsa	2361	3	1	0
31_GB_1_1_1	Samastipur	2	1	1	1
31_GB_1_2_4	Samastipur	2	3	1	1
31_GB_1_3_5	Samastipur	257	4	1	1
31_GB_1_4_6	Samastipur	2	3	1	0
31_GB_2_1_1	Samastipur	1162	1	1	1
31_GB_2_2_5	Samastipur	244	4	1	1
31_GB_2_3_6	Samastipur	1227	3	1	0
31_MB_3_1_1	Samastipur	2	1	1	1
31_MB_3_2_5	Samastipur	186	4	1	1
31_MB_4_1_1	Samastipur	2	1	1	1
31_MB_4_2_6	Samastipur	2	3	1	0
32_PH_3_1_1	Begusarai	23	1	1	1
32_PH_3_2_4	Begusarai	5350	3	1	1
32_PH_4_1_2	Begusarai	2	2	1	1
32_PH_4_2_4	Begusarai	1759	3	1	1
32_SC_1_1_1	Begusarai	2	1	1	1
32_SC_1_2_4	Begusarai	1790	3	1	1
32_SC_1_3_5	Begusarai	2	4	1	1
32_SC_2_1_1	Begusarai	2	1	1	1
32_SC_2_2_4	Begusarai	1245	3	1	1
32_SC_2_3_5	Begusarai	2	4	1	1
33_LP_1_1_2	Bhagalpur	2	2	1	1
33_LP_1_2_4	Bhagalpur	2	3	1	1
33_LP_2_2_4	Bhagalpur	2	3	1	1
33_SC_3_1_2	Bhagalpur	2	2	1	0
33_SC_3_2_4	Bhagalpur	2	3	1	0
33_SC_4_1_1	Bhagalpur	105	1	1	1
33_SC_4_2_4	Bhagalpur	2	3	1	1
33_SC_5_2_4	Bhagalpur	2	3	1	0
34_GB_1_1_1	Chappra	2	1	1	1
34_GB_2_1_1	Chappra	2	1	1	1
34_GB_2_2_4	Chappra	1638	3	1	1
34_GM_3_1_1	Chappra	2312	1	1	1
34_GM_3_2_4	Chappra	2043	3	1	1
34_MC_4_1_1	Chappra	2	1	1	0
34_MC_4_2_4	Chappra	3006	3	1	0
34_MC_5_1_1	Chappra	2	1	1	1
34_MC_5_2_4	Chappra	1929	3	1	1

34_MC_5_3_4	Chappra	1491	3	1	1
35_LB_3_1_1	Betia	2804	1	1	0
35_LB_3_2_4	Betia	160	3	1	0
35_LB_4_1_1	Betia	759	1	1	0
35_LB_4_2_4	Betia	2579	3	1	0
35_MB_1_1_1	Betia	2	1	1	0
35_MB_1_2_4	Betia	825	3	1	0
35_MB_1_3_5	Betia	2	4	1	0
35_MB_2_1_1	Betia	558	1	1	0
35_MB_2_2_4	Betia	3507	3	1	0
35_MB_2_3_5	Betia	2	4	1	0
36_GB_1_1_1	Buxar	305	1	1	1
36_GB_2_2_4	Buxar	2766	3	1	1
36_MC_3_1_1	Buxar	6416	1	1	1
36_MC_3_2_4	Buxar	2029	3	1	1
36_MC_3_3_4	Buxar	1425	3	1	1
36_MC_4_2_1	Buxar	2	1	1	1
36_MC_4_3_4	Buxar	321	3	1	1
37_CP_1_1_1	Gaya	3177	1	1	1
37_CP_1_2_4	Gaya	1220	3	1	1
37_CP_1_3_6	Gaya	2	3	1	1
37_CP_2_1_1	Gaya	970	1	1	1
37_CP_2_2_4	Gaya	3648	3	1	1
37_PG_3_1_1	Gaya	23	1	1	1
37_PG_3_2_4	Gaya	5107	3	1	1
37_PG_4_1_1	Gaya	320	1	1	1
37_PG_4_2_4	Gaya	1520	3	1	1
37_PG_4_3_7	Gaya	2	4	1	1
38_GD_1_1_1	Varanasi	2	1	0	1
38_GD_1_2_4	Varanasi	1355	3	0	1
38_GD_2_2_4	Varanasi	4320	3	0	1
38_VG_3_1_1	Varanasi	847	1	0	1
38_VG_3_2_4	Varanasi	1763	3	0	1
38_VG_4_1_1	Varanasi	2330	1	0	1
38_VG_4_2_4	Varanasi	2061	3	0	1
38_VG_5_1_1	Varanasi	931	1	0	1
38_VG_5_2_4	Varanasi	2022	3	0	1
39_KP_4_1_1	Gorakhpur	1191	1	0	1
39_KP_4_2_4	Gorakhpur	94	3	0	1
39_KP_5_1_4	Gorakhpur	135	3	0	1

39_KP_5_2_4	Gorakhpur	3535	3	0	1
39_SB_2_1_1	Gorakhpur	25	1	0	1
39_SB_2_2_4	Gorakhpur	3522	3	0	1
39_SG_1_1_1	Gorakhpur	73	1	0	1
39_SG_1_2_4	Gorakhpur	1524	3	0	1
39_SG_3_1_1	Gorakhpur	1588	1	0	1
39_SG_3_2_4	Gorakhpur	4121	3	0	1
39_SG_3_3_6	Gorakhpur	2	3	0	1
40_RP_3_1_1	Daltonganj	2	1	0	0
40_RP_3_2_4	Daltonganj	2321	3	0	0
40_RP_4_1_1	Daltonganj	34	1	0	0
40_RP_4_2_4	Daltonganj	3117	3	0	0
40_RP_5_1_1	Daltonganj	2	1	0	1
40_RP_5_2_4	Daltonganj	3291	3	0	1
40_SM_1_1_1	Daltonganj	716	1	0	0
40_SM_1_2_4	Daltonganj	2766	3	0	0
40_SM_2_1_1	Daltonganj	15	1	0	0
40_SM_2_2_4	Daltonganj	2124	3	0	0
41_GR_3_1_1	Hazaribagh	32	1	0	0
41_GR_3_2_4	Hazaribagh	1941	3	0	0
41_GR_4_1_1	Hazaribagh	2497	1	0	0
41_GR_4_2_4	Hazaribagh	4205	3	0	0
41_SM_1_1_1	Hazaribagh	2968	1	0	1
41_SM_1_2_4	Hazaribagh	1272	3	0	0
41_SM_1_3_6	Hazaribagh	2	3	0	1
41_SM_2_1_1	Hazaribagh	1887	1	0	0
41_SM_2_2_4	Hazaribagh	1094	3	0	0
41_SM_2_3_6	Hazaribagh	2	3	0	1
42_DT_4_1_1	Deoghar	192	1	0	1
42_DT_4_2_4	Deoghar	2	3	0	1
42_DT_5_1_1	Deoghar	82	1	0	1
42_DT_5_2_4	Deoghar	2256	3	0	1
42_MB_1_1_1	Deoghar	332	1	0	1
42_MB_1_2_4	Deoghar	5950	3	0	1
42_MB_2_1_1	Deoghar	1454	1	0	1
42_MB_2_2_4	Deoghar	2	3	0	1
42_MB_3_1_2	Deoghar	240	2	0	1
42_MB_3_2_4	Deoghar	469	3	0	1
43_KB_1_1_1	Brahmpore	1886	1	0	0
43_KB_1_2_5	Brahmpore	2	4	0	0

43_KB_2_1_2	Brahmpore	54	2	0	0
43_KB_2_2_4	Brahmpore	1204	3	0	0
43_NB_3_1_1	Brahmpore	2054	1	0	0
43_NB_3_2_4	Brahmpore	143	3	0	0
43_NB_4_1_1	Brahmpore	2629	1	0	0
43_NB_4_2_4	Brahmpore	2	3	0	0
43_NB_5_1_1	Brahmpore	2662	1	0	0
43_NB_5_2_4	Brahmpore	562	3	0	0
44_AB_1_2_5	Malda	2	4	0	0
44_AB_2_1_1	Malda	2	1	0	0
44_AB_2_2_5	Malda	2	4	0	0
44_RB_3_2_2	Malda	2	2	0	0
44_RB_4_1_1	Malda	2	1	0	0
44_RB_4_2_5	Malda	2	4	0	0
44_RB_5_1_1	Malda	2	1	0	0
44_RB_5_2_5	Malda	2	4	0	0
45_LC_6_1_4	Ranchi	2220	3	0	
45_LC_6_2_1	Ranchi	2	1	0	
45_LC_7_1_4	Ranchi	4	3	0	
45_LC_7_2_3	Ranchi	2	2	0	
45_LC_7_3_3	Ranchi	2	2	0	
45_UB_1_1_4	Ranchi	2	3	0	1
45_UB_1_2_3	Ranchi	2	2	0	1
45_UB_2_1_4	Ranchi	2	3	0	1
45_UB_2_2_4	Ranchi	2432	3	0	1
45_UB_3_1_4	Ranchi	2786	3	0	1
45_UB_3_2_1	Ranchi	173	1	0	1
45_UB_4_1_4	Ranchi	2343	3	0	1
45_UB_4_2_4	Ranchi	2	3	0	1
45_UB_4_3_1	Ranchi	2	1	0	1
45_UB_5_1_4	Ranchi	1899	3	0	1
45_UB_5_2_4	Ranchi	1867	3	0	1
45_UB_5_3_4	Ranchi	8	3	0	1
45_UB_5_4_1	Ranchi	2	1	0	1
45_UB_5_5_1	Ranchi	2	1	0	1
46_RM_1_1_1	Jamshedpur	2	1	0	1
46_RM_1_2_4	Jamshedpur	2	3	0	1
46_RM_1_3_4	Jamshedpur	2	3	0	1
46_RM_1_4_4	Jamshedpur	2	3	0	1
46_RM_2_1_2	Jamshedpur	3	2	0	1

46_RM_2_2_4	Jamshedpur	2	3	0	1
46_RM_2_3_4	Jamshedpur	2	3	0	1
46_RM_3_1_2	Jamshedpur	2	2	0	1
46_RM_3_2_2	Jamshedpur	2	2	0	1
46_RM_3_3_4	Jamshedpur	2	3	0	1
46_RM_3_4_4	Jamshedpur	2	3	0	1
46_RM_4_1_4	Jamshedpur	2	3	0	
46_RM_4_2_2	Jamshedpur	130	2	0	
46_RM_5_1_1	Jamshedpur	2	1	0	
46_RM_5_2_4	Jamshedpur	2	3	0	
47_MB_1_1_1	Asansol	2	1	0	
47_MB_1_2_4	Asansol	2	3	0	
47_MB_2_1_1	Asansol	2	1	0	1
47_MB_2_2_4	Asansol	2	3	0	1
47_MB_3_1_4	Asansol	2	3	0	
47_MB_3_2_4	Asansol	2	3	0	
47_MB_4_1_1	Asansol	2	1	0	
47_MB_4_2_2	Asansol	2	2	0	
47_SM_5_1_1	Asansol	2	1	0	
47_SM_5_2_4	Asansol	2	3	0	
47_SM_5_3_4	Asansol	2	3	0	
47_SM_6_1_1	Asansol	2	1	0	
47_SM_6_2_4	Asansol	2	3	0	
48_BC_1_1_4	Durgapur	2	3	0	
48_BC_1_1_5	Durgapur	2	4	0	
48_BC_2_1_1	Durgapur	2	1	0	0
48_BC_2_2_4	Durgapur	2	3	0	0
48_BC_2_3_1	Durgapur	2	1	0	0
48_SM_3_1_3	Durgapur	2	2	0	0
48_SM_3_2_1	Durgapur	2	1	0	1
48_SM_4_1_1	Durgapur	3	1	0	0
48_SM_4_2_1	Durgapur	2	1	0	0
49_MD_4_1_1	Bardhman	2	1	0	
49_MD_5_1_1	Bardhman	2	1	0	
49_NG_1_1_3	Bardhman	2	2	0	0
49_NG_1_2_4	Bardhman	2	3	0	1
49_NG_2_1_2	Bardhman	2	2	0	1
49_NG_2_2_4	Bardhman	2	3	0	1
49_NG_3_1_2	Bardhman	2	2	0	1
49_NG_3_2_4	Bardhman	2	3	0	1

50_AG-1-1-5	Ambikapur	2	4	0	
50_AG-1-2-6	Ambikapur	1114	3	0	
50_AG-1-3-7	Ambikapur	8	4	0	
50_BR-1-1-1	Ambikapur	2	1	0	
50_GD-1-1-1	Ambikapur	2	1	0	
50_GD-1-2-1	Ambikapur	210	1	0	
50_KD-1-1-4	Ambikapur	961	3	0	
50_KD-1-2-5	Ambikapur	10	4	0	
50_KD-1-3-6	Ambikapur	2	3	0	
50_KD-1-4-7	Ambikapur	2	4	0	
50_KD-1-5-8	Ambikapur	3125	3	0	
50_KM-1-1-3	Ambikapur	2	2	0	
50_MT-1-1-4	Ambikapur	2	3	0	
50_MT-1-2-5	Ambikapur	2	4	0	
50_MT-1-3-9	Ambikapur	2	4	0	
50_SD-1-1-1	Ambikapur	2	1	0	
50_SR-1-1-3	Ambikapur	2	2	0	
50_SR-1-2-5	Ambikapur	2	4	0	
51_VV_1_1_1	Bilaspur	2	1	0	0
51_VV_1_2_4	Bilaspur	2	3	0	0
51_VV_1_3_5	Bilaspur	2	4	0	0
51_VV_1_4_6	Bilaspur	2	3	0	0
51_VV_1_5_7	Bilaspur	2	4	0	0
51_VV_2_1_1	Bilaspur	2	1	0	1
51_VV_2_2_4	Bilaspur	3491	3	0	1
51_VV_2_3_5	Bilaspur	16	4	0	1
51_VV_2_4_6	Bilaspur	3383	3	0	1
51_VV_2_5_7	Bilaspur	5255	4	0	1
51_VV_2_6_3	Bilaspur	2	2	0	0
51_VV_3_1_1	Bilaspur	2	1	0	0
51_VV_3_2_6	Bilaspur	2	3	0	0
51_VV_3_3_9	Bilaspur	2	4	0	0
52_AG_1_1_8	Aligarh	2	3	0	
52_AG_1_2_6	Aligarh	2	3	0	1
52_AG_1_3_1	Aligarh	3	1	0	1
52_AG_1_4_1	Aligarh	2	1	0	1
52_AG_2_1_1	Aligarh	22	1	0	1
52_AG_2_2_1	Aligarh	13	1	0	1
52_AG_3_1_5	Aligarh	2	4	0	1
52_AG_3_2_6	Aligarh	2	3	0	1

52_AG_4_1_5	Aligarh	2	4	0	1
52_AG_4_2_6	Aligarh	2	3	0	1
52_AG_4_3_1	Aligarh	2	1	0	1
52_AG_4_4_1	Aligarh	2	1	0	1
52_AG_5_1_5	Aligarh	2	4	0	1
52_AG_5_2_6	Aligarh	2	3	0	1
52_AG_5_3_2	Aligarh	2	2	0	1
53_GN_1_1_1	Kanpur	2	1	0	0
53_GN_1_2_5	Kanpur	2	4	0	0
53_GN_2_1_4	Kanpur	2	3	0	
53_GN_2_2_6	Kanpur	2	3	0	
53_GN_3_1_1	Kanpur	2	1	0	
53_GN_3_2_4	Kanpur	2	3	0	
53_GN_3_3_6	Kanpur	2	3	0	
53_NG_1_1_1	Kanpur	2	1	0	1
53_NG_1_1_6	Kanpur	2	3	0	1
53_NG_1_2_1	Kanpur	2	1	0	0
53_NG_1_2_4	Kanpur	2	3	0	1
53_NG_1_3_5	Kanpur	2	4	0	
53_NG_1_4_6	Kanpur	2	3	0	
53_NG_1_5_7	Kanpur	2	4	0	
53_NG_2_1_1	Kanpur	2	1	0	0
53_NG_2_1_1	Kanpur	2	1	0	1
53_NG_2_2_4	Kanpur	2	3	0	1
53_NG_2_2_5	Kanpur	2	4	0	1
53_NG_2_3_4	Kanpur	2	3	0	1
53_NG_2_3_5	Kanpur	2	4	0	1
53_NG_2_4_6	Kanpur	2	3	0	1
53_NG_2_5_7	Kanpur	2	4	0	1
53_NG_3_1_1	Kanpur	2	1	0	0
53_NG_3_1_1	Kanpur	2	1	0	0
53_NG_3_2_4	Kanpur	2	3	0	1
53_NG_3_2_5	Kanpur	2	4	0	1
53_NG_3_3_5	Kanpur	2	4	0	1
53_NG_3_3_6	Kanpur	2	3	0	1
53_NG_3_4_7	Kanpur	2	4	0	1
53_NG_4_1_4	Kanpur	2	3	0	
53_NG_4_2_3	Kanpur	2	2	0	
53_NG_4_3_6	Kanpur	2	3	0	
53_NG_4_4_7	Kanpur	2	4	0	

54_RN_1_1_1	Prayagraj	2	1	0	0
54_RN_1_2_5	Prayagraj	2	4	0	0
54_RN_1_3_6	Prayagraj	2	3	0	0
54_RN_2_1_1	Prayagraj	2	1	0	0
54_RN_2_2_5	Prayagraj	2	4	0	0
54_RN_2_3_6	Prayagraj	2	3	0	0
54_RN_3_1_1	Prayagraj	2	1	0	0
54_RN_3_2_5	Prayagraj	2	4	0	0
54_RN_3_3_6	Prayagraj	2	3	0	0
54_SM_1_1_1	Prayagraj	2	1	0	
54_SM_1_1_1	Prayagraj	2	1	0	0
54_SM_1_2_2	Prayagraj	2	2	0	1
54_SM_1_2_4	Prayagraj	2	3	0	
54_SM_1_3_4	Prayagraj	2	3	0	1
54_SM_1_3_6	Prayagraj	2	3	0	
54_SM_1_4_6	Prayagraj	4	3	0	1
54_SM_2_1_1	Prayagraj	2	1	0	
54_SM_2_2_4	Prayagraj	2	3	0	0
54_SM_2_3_6	Prayagraj	2	3	0	0
54_UB_1_1_1	Prayagraj	2	1	0	
54_UB_1_2_2	Prayagraj	2	2	0	
54_UB_1_3_4	Prayagraj	2	3	0	
54_UB_1_4_6	Prayagraj	2	3	0	
54_UB_3_1_1	Prayagraj	2	1	0	
54_UB_3_2_4	Prayagraj	2	3	0	
54_UB_3_3_5	Prayagraj	2	4	0	
54_UB_3_4_6	Prayagraj	2	3	0	
54_UD_1_1_1	Prayagraj	2	1	0	0
54_UD_1_1_1	Prayagraj	2	1	0	0
54_UD_1_2_4	Prayagraj	2	3	0	0
54_UD_1_3_6	Prayagraj	2	3	0	0
54_UD_3_1_1	Prayagraj	2	1	0	0
54_UD_3_2_4	Prayagraj	2	3	0	0
54_UD_3_3_5	Prayagraj	2	4	0	1
54_UD_3_4_5	Prayagraj	2	4	0	
54_UD_3_5_6	Prayagraj	2	3	0	
55_CB_1_1_1	Bahraich	2	1	0	0
55_CB_1_1_1	Bahraich	2	1	0	0
55_CB_1_2_4	Bahraich	2	3	0	0
55_CB_1_2_4	Bahraich	2	3	0	0

55_CB_1_3_5	Bahraich	2	4	0	0
55_CB_1_4_6	Bahraich	2	3	0	0
55_CB_2_1_1	Bahraich	2	1	0	0
55_CB_2_2_4	Bahraich	2	3	0	0
55_CB_2_3_5	Bahraich	2	4	0	0
55_CB_2_4_6	Bahraich	2	3	0	0
55_CB_3_1_1	Bahraich	2	1	0	0
55_CB_3_2_4	Bahraich	2	3	0	0
55_CB_3_3_6	Bahraich	2	3	0	0
55_CL_4_1_1	Bahraich	2	1	0	0
55_CL_4_2_4	Bahraich	2	3	0	0
55_CL_4_3_5	Bahraich	2	4	0	0
55_CL_4_4_6	Bahraich	2	3	0	0
55_CL_5_1_1	Bahraich	2	1	0	0
55_CL_5_2_4	Bahraich	2	3	0	0
55_CL_5_3_7	Bahraich	2	4	0	0
55_CL_5_4_6	Bahraich	2	3	0	0
55_CL_6_1_1	Bahraich	2	1	0	0
55_CL_6_2_4	Bahraich	2	3	0	0
55_CL_6_3_6	Bahraich	2	3	0	0
55_DK_10_1_1	Bahraich	2	1	0	0
55_DK_10_2_4	Bahraich	2	3	0	0
55_DK_10_3_5	Bahraich	2	4	0	0
55_DK_10_4_6	Bahraich	2	3	0	0
55_DK_11_1_1	Bahraich	2	1	0	0
55_DK_11_2_4	Bahraich	2	3	0	0
55_DK_11_3_6	Bahraich	2	3	0	0
55_DK_12_1_1	Bahraich	2	1	0	0
55_DK_12_2_6	Bahraich	2	3	0	0
55_GG_7_1_1	Bahraich	2	1	0	0
55_GG_7_2_4	Bahraich	2	3	0	0
55_GG_7_3_5	Bahraich	2	4	0	0
55_GG_7_4_6	Bahraich	2	3	0	0
55_GG_8_1_1	Bahraich	2	1	0	0
55_GG_8_2_4	Bahraich	2	3	0	0
55_GG_8_3_5	Bahraich	2	4	0	0
55_GG_8_4_6	Bahraich	2	3	0	0
55_GG_9_1_1	Bahraich	2	1	0	0
55_GG_9_2_4	Bahraich	2	3	0	0
55_GG_9_3_5	Bahraich	2	4	0	0

55_GG_9_4_6	Bahraich	2	3	0	0
5_MM_1_1_1	Patna	1228	1	1	1
5_MM_1_2_4	Patna	971	3	1	1
5_MM_1_3_7	Patna	2	4	1	1
5_MM_2_1_1	Patna	1959	1	1	1
5_MM_2_2_4	Patna	2	3	1	1
5_MM_2_3_6	Patna	2	3	1	1
5_MM_3_1_1	Patna	3519	1	1	1
5_MM_3_2_4	Patna	2961	3	1	1
5_MM_3_3_6	Patna	2	3	1	1
5_MM_4_1_4	Patna	2126	3	1	1
5_MM_4_2_5	Patna	2	4	1	1
5_MM_5_1_6	Patna	2	3	1	1
5_MM_5_2_4	Patna	1726	3	1	1
5_MM_5_3_5	Patna	2	4	1	1
5_MM_6_1_6	Patna	2	3	1	
5_MM_6_2_4	Patna	2456	3	1	
6_BB_1_1_2	Kolkata	2	2	0	1
6_BB_1_2_1	Kolkata	6	1	0	1
6_BB_1_3_1	Kolkata	2	1	0	1
6_BB_2_1_1	Kolkata	2	1	0	1
6_BB_2_2_4	Kolkata	2	3	0	1
6_BB_3_1_1	Kolkata	2	1	0	1
6_BB_3_2_1	Kolkata	2	1	0	1
6_BB_3_3_4	Kolkata	2	3	0	1
6_BB_4_1_1	Kolkata	2	1	0	1
6_RB_5_1_4	Kolkata	2	3	0	
6_RB_5_2_3	Kolkata	2	2	0	0
6_RB_6_1_1	Kolkata	2	1	0	

162
163
164

Turmeric wholesale/vendor/businessperson interview guide

Demographic information: Age, sex, religion, education level, primary occupation/title, years involved in business

Before visiting the market check is there a specific day when the market is closed? What's a good time to visit? Identify a time when there's less rush, so the vendor will be free to talk.

Once you arrive at the market. Walk around and locate a few wholesale vendors selling different types of turmeric. Different types refers to turmeric in whole/processed forms and turmeric grown/polished in different places.

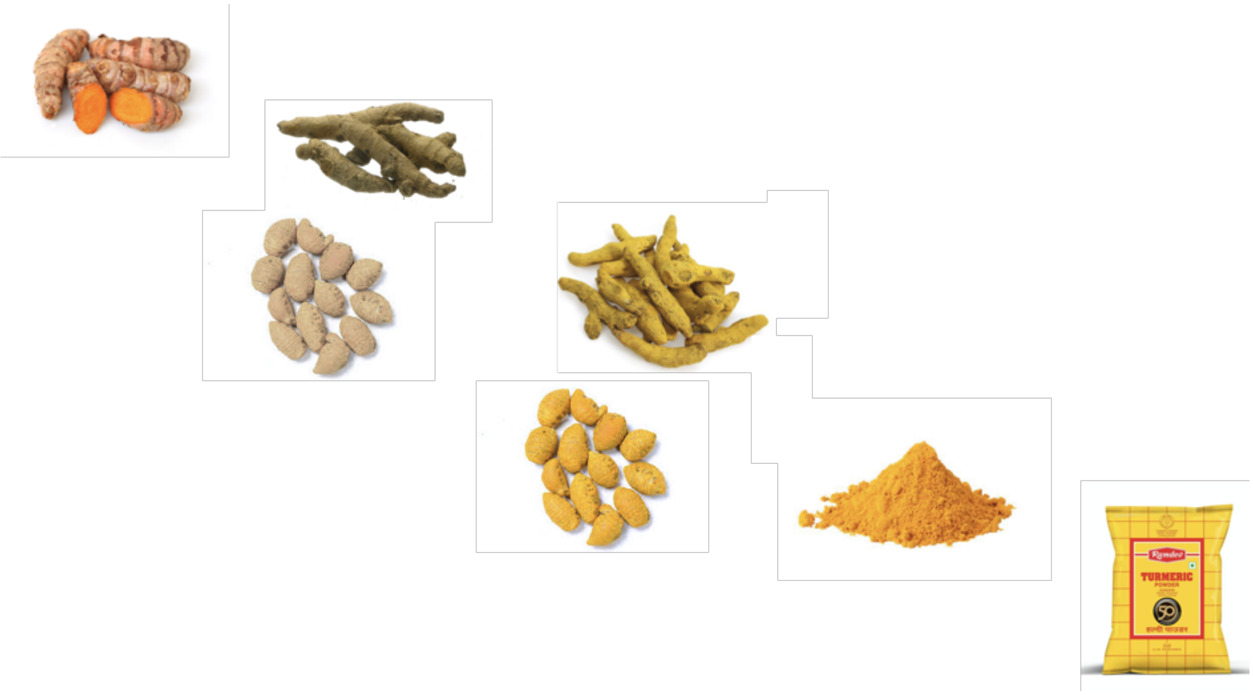

Figure 1. Examples of different forms of turmeric from left to right (fresh root, dried unpolished finger roots and bulbs, dried polished finger roots and bulbs, loose powder, and packaged branded powder.

A. **Market-level questions**

- Date
- Interviewer
- City name
- Market type (wholesale or retail)
- Total number of spice vendors in market (approximate)

- Major supplying regions to the market (e.g., where is turmeric coming from – the state/district of harvest/notable suppliers)?
- Major distribution regions from the market (e.g., where is turmeric being distributed to from the wholesalers?)
- Other notable market features – predominantly what commodity types/etc.?

B. **Vendor-level questions**

- Vendor number
- When is turmeric season?
- Types of turmeric for sale – for each, state where the spice was harvested.

1 Loose Powder
2 Packaged Powder (no brand)
3 Packaged branded powder
4 Dried polished finger
5 Dried unpolished finger
6 Dried polished bulb
7 Dried unpolished bulb
8 Mix of dried finger and bulb (polished)
9 Mix of dried finger and bulb (unpolished)
10 Fresh root (finger or bulb)
11. Loose chili powder
12. Packaged chilli powder (no brand)
13. Loose dried chili
14. Packaged dried chili (no brand)
15. Loose coriander powder
16. Packaged coriander powder (no brand)
17. Loose dried coriander
18. Packaged dried coriander (no brand)

- If packaged branded powder was selected, please write the names of the brands.
- How do your purchasing patterns vary throughout the year? For example, do you purchase from different people/locations in turmeric season and out of season? If yes, who do you primarily purchase from in season and out of season?
- Commodities sold by vendor - other than turmeric
- Does the vendor do their own grinding? (if powder being being sold) If yes, ask “grinder interview questions”). If no, determine where grinding occurs.
- Does the vendor do their own polishing? If yes, ask “polisher interview questions”).
- How much turmeric do you sell per year (in kg/tn)?

in-season? out-of-season?

- Customers: Who are the vendor's main customers? (Probe about which are primary customers, if any).

1 Companies or distributors
2 Wholesale vendors
3 Retail vendors
4 Street vendors

5 High-end restaurant/hotels
6 Low-end restaurant/hotels
7 Higher income household customers
8 Lower income household customers

- What is the geographic extent of the vendor's turmeric customers? (local domestic market, broader/regional domestic market, international/export market)

Suppliers

- Does the vendor have one fixed supplier or is it variable? In snowball sampling fashion, seek information about suppliers to determine if possible to interview them.
- Does the same person/entity supply the entire market?
- Does the vendor have a direct link to the turmeric polisher and/or farmer? In snowball sampling fashion, seek information about suppliers to determine if possible to interview them.
- Are there ever shortages of certain types of turmeric of different types throughout the year? If yes, which and why?

Quality

- In your opinion, where does turmeric of the best quality come from in India? What about turmeric of the lowest quality?
- How does the vendor check for turmeric quality? (For example, breaking open a root to look at inner color, inner oil content, etc. Probe about differences in checking for quality based on turmeric form: turmeric powder, dried finger root, or dried bulb root, etc.)
- If they check for quality, probe further to ask about what the vendor is looking for. Specifically, what attributes make turmeric high quality? For example, they might break the roots into pieces and look for the inner color and oil content which they can feel on their fingers or they might even cook with the turmeric powder they might check for quality by the color of the resulting dish (curry) and also check to be sure the taste is good with minimal bitterness. OR if they only have dried bulb roots, they may not be able to break them open and check for any quality except what their finger can scratch off.
- Does the vendor get the quality tested personally? (What specifically is being tested for - e.g., curcumin? Where do they get it tested - e.g., government lab? What motivated them to get the turmeric tested?)
- Are there any threats to quality? Any added substances to improve colour or other traits? (Probe about differences in threats to quality if the turmeric is powder, dried finger root, or dried bulb root, etc.) (For example, if turmeric is in powder form, it is easy to add flour or other filler substances.)

Additives

- Some people we have talked about adding color/pigments to turmeric, what have you heard about this?
    - How widespread is the practice across the country of India – does it occur more in some states/districts than others? Why?
    - Who in the supply chain adds color?

- When and why did people start adding color in this region (specify district if mentioned)?
- What factors affect if people add color from year-to-year? What factors impact how much color is added or how frequently it is added?
  - Probe about:
    1. Type: Fingers vs. bulbs
    2. Production origin: Bihar vs. Jharkhand vs. Tamil Nadu etc.
    3. Destination: Turmeric for local markets versus large wholesale markets in cities in the district
    4. Turmeric crop quantity/quality of the year (e.g., climate affecting the yield/quantity of the turmeric crop)?
    5. Who, if anyone, asks for the addition of color?
    6. Why do they ask for color to be added?
    7. Do they ask for color to be added to all types of turmeric (finger vs. bulb from different regions) that are processed or only to certain types?
    8. Who decides to add the color? (Mill owner, operator,etc.)
    9. Is there any reason that color wouldn't be added if it is requested?

Inspections

- Does anyone ever do any inspections of your business? Who does inspections? What are they inspecting for? How frequently do they do inspections? What are the repercussions/penalties for not passing an inspection?
- Are there any punishments for other shops that sell adulterated products? If yes, what? (Probe specifically about turmeric and type of adulterant)

Turmeric grinder interview guide

Demographic information: Age, sex, religion, education level, primary occupation/title, years involved in business

<u>Observations</u>

- Where is the grinding business situated (in a market, on its own…)?
- What other businesses/approximately how many are present at the location? (e.g., in a market of 20 vendors selling spices, grains, and dried goods)
- How many other grinding businesses are present at the location?
- What is the condition of the grinding stone – cracked/filled with solder, uncracked in good condition, etc.?
- What is currently being ground/what appears to have been ground recently?
- Visible color/pigments being used – what hue/what type

<u>Questions</u>

- Describe your daily operations – what you do from the moment you open your business to the moment you close your business on a typical day.
- Do you face any challenges in the operation of your business? If yes, what?
- Who are your customers? (e.g., vendors selling ground goods in the market, companies grinding spice or grains for package/sale elsewhere, or individuals grinding small quantities)
- What foods/materials do you grind? Spices, grains, etc.
- Between food types, do you do anything to clean or ready your machinery for the next item? For example, let's say you were just grinding turmeric root and now you need to grind
- What rates do you charge for grinding materials (e.g., per kg ground)? Do your rates of grinding cost per kg vary by food type? Do rates vary by quantity ground? Do you have a minimum amount (e.g., X kg) required for grinding?
- Do you ever have to repair your grinding equipment/grinding stone? If yes, how do you do that?
- Does anyone ever do any inspections of your business? Who does inspections? What are they inspecting for? How frequently do they do inspections? What are the repercussions/penalties for not passing an inspection?
- Are there any punishments for other shops that sell adulterated products? If yes, what?

Turmeric polisher interview guide

Demographic information: Age, Sex, religion, education level, primary occupation/title, years involved in business

Directions: Aim to speak with two individuals per polishing mill: 1) the owner, and 2) a worker who operates the polishing machine. Ideally, send two interviewers so one person can speak with the owner at the same time as the other person is speaking with the polishing machine operator. The reason for this is that sometimes a worker will share more information more honestly when the owner is preoccupied.

Observations

- Move around the processing mill and observe is there any empty packet of yellow pigment. *At the end only, after* completing the data collection, ask the following about the empty packet (may not answer these questions):
    - What is it? What is the local name of whatever was inside?
    - What is the purpose of using it? Why?
    - How do they use it?
    - From where they bring it?
    - What is the price?

Questions

Polisher ID – Assign a unique 2-digit polisher ID to the polisher starting with 01 and advancing.

Date and time of visit

Mill location – district, sub-district, village, GPS coordinates

- Describe your daily operations – what you do from the moment you open your business to the moment you close your business on a typical day.
- Do you face any challenges in the operation of your business? If yes, what?

# Years Active or Inactive

- Number of years the polishing mill has been active and inactive. For example, a mill may have been inactive for the past 2 years, but active for 5 years before that.

Number of polishing machines and the capacity in kg for each machine

- What is the maximum capacity or size of the machine(s)? For example, how many kg, or tons (tn) of roots can fit in the machine for polishing at a time? Write separately for each machine and add notes or additional machines as necessary.

Seasonality

- What are the typical months when turmeric is harvested, dried, and polished? What are you busiest months of operation? What are your slowest months?

Employees

- Write the number of employees year-round and any additional employees in turmeric season. The sum of these numbers should be the total number of employees.

Quantity polished

- Write the total amount (kg/tons) of turmeric you polish in turmeric season AND outside of turmeric season. Write total and whether it is in kg, or tons).

Customers

- To what cities/locations is your polished turmeric distributed? List them and mention relative proportions – e.g., 50% to Bihar, 25% to Buxar, 25% to Varanasi.
- Write any contact information for customers/destination of turmeric (write in order from those who you sell the most turmeric to, to those who you sell the least by volume).

Suppliers

- Who supplies your turmeric throughout the year (probe about differences in turmeric season and outside of turmeric season)? Where is the turmeric grown and dried (note the name of the district/village as well as the approximate distance from the polishing mill).

Quality

- In your opinion, where does turmeric of the best quality come from in India? What about turmeric of the lowest quality?

Additives

- We have heard that some people add additives to turmeric to enhance its color or quantity. What do you know about this?
- Some people have mentioned that grinding mills sometimes add rice flour or other starchy substances to turmeric powder. What do you know about this?
- Some polishing mills have mentioned that additives like colored powders are added to enhance turmeric color during polishing. What do you know about this practice?
- Write name of substances you know about, cost (rupees/kg), and amount added (g or kg of color per kg turmeric). Note if different for finger or bulb. Does your mill ever add these? If so, why?

Inspection

Some people have told us that their mills have been inspected by police/the government. Has your mill ever been inspected? How frequently? When was the last inspection? What do inspectors inspect for? Do inspectors care if you are adding color?

Turmeric farmer interview guide

Demographic information: Age, sex, religion, education level, primary occupation/title, years involved in business

Questions

Date and time of visit

Location – district, sub-district, village

- Describe your daily operations – what you do from the moment you wake up to the moment you go to sleep on a typical day. Do you only grow turmeric or also dry, and polish it?
- Do you face any challenges in the operation of your business? If yes, what?
- What other crops do you grow on your land? Is there a rotation during different months
- Area of land for growing turmeric

Quantity/varieties grown

- Write the total amount (kg/tons of fresh or dried root) of turmeric you harvest?
- Where do you get your turmeric seeds? How many varieties do you grow?

Turmeric season

- During what months do you sow seeds?
- During what months do you harvest turmeric root?
- During what months do you dry turmeric root?

Employees

- Write the number of employees year-round and any additional employees in turmeric season. The sum of these numbers should be the total number of employees.

Customers

- To what cities/locations is your turmeric distributed? List them and mention relative proportions – e.g., 50% to Bihar, 25% to Buxar, 25% to Varanasi.

Quality

- In your opinion, where does turmeric of the best quality come from in India? What about turmeric of the lowest quality?

Additives

- We have heard that some people add additives to turmeric to enhance its color or quantity. What do you know about this?
- Some people have mentioned that grinding mills sometimes add rice flour or other starchy substances to turmeric powder. What do you know about this?
- Some polishing mills have mentioned that additives like colored powders are added to enhance turmeric color during polishing. What do you know about this practice?
- Write name of substances you know about, cost (rupees/kg), and amount added (g or kg of color per kg turmeric). Note if different for finger or bulb.

Inspection

- Some people have told us that polishing mills have been inspected by police/the government. What do you know about this? What do inspectors look for? Do they care if you people are adding color?

Food safety authority/inspector/police interview guide
Demographic information: Age, sex, religion, education level, primary occupation/title, years involved in business

Information of Job responsibilities
- Who is your employer – name of agency? Who do you report to? Who reports to you?
- Describe your job responsibilities
- Do you conduct food safety inspections? If yes, what types of places and facilities do you inspect? What are you looking for? (Try to understand which types of issues are most to least important) How frequently do you inspect different types of food places? Who accompanies you during inspection?
- What are the repercussions for failing an inspection? If you find a food safety violation and someone fails an inspection, after how many days do you revisit the same place?
- Do you do any inspections related to spices? If yes, how frequently? Please follow-up with the same questions as above.
- Have you ever conducted an inspection of a spice grinding facility? When? What were you looking for?
- When was the last time you encountered a food safety violation related to spices. What spice? What was the problem? What happened?

Questions regarding turmeric
- Have you ever conducted an inspection of a turmeric polishing mill? When? What were you looking for?
- What kind of adulteration have you found in turmeric? (probe about specifics as sometimes vague language can be used)
- What is the process of identifying adulteration? How long does it take/costs involved?
- What is the action you take if you find turmeric adulteration – probe about adulteration via starchy substances adding volume versus yellow pigments adding color and when in they are added to root and/or powder?
- What is the punishment for adulteration?
- Have you experienced any difficulties/barriers dealing with [those people]? If yes, what kind? Please explain.
- (If not mentioned earlier) Did you hear about lead chromate yellow industrial pigments being added to spices like turmeric? Probe about specifics – added to root or powder, etc.
    - Why do you think this turmeric adulteration occurs? Where do you think it occurs? Who is responsible?
    - What could be done to stop spice adulteration like that of turmeric?
- What could be done to make stronger to the inspection team?

Yellow pigment wholesaler/vendor/businessperson interview guide

Demographic information: Age, sex, religion, education level, primary occupation/title, years involved in business

- What pigments do you sell? How many types of yellow pigments do you sell and what are their names? note colors of pigment powders available for sale, where the pigments are produced/imported from, and the cost of each unique pigment type
- Who do you sell your pigments to and what do they do with them?
- What are your most popular pigments? (note them by color/name)
- For each type of yellow pigment…
    - How long have you sold it?
    - Where do you get it from?
    - Is it locally produced or imported (if imported, from where?)
    - What do people buy it for?
    - How does it rank in terms of popularity compared to other types of yellow pigments?
    - How much does it cost per kg?
    - In what form is it sold (e.g., loose, packaged, dry, mixed as paint, or all)?
    - Who are your primary customers (what do they do with it – be specific)?
    - How many kg do you sell per month/year?
    - Are any of your pigments safe for adding to foods for consumption?
    - Are there any regulations or restrictions on your pigments of any color (note which colors). If yes, when were they established, how are they enforced, what do they intend to do?

Description of qualitative methods

Prior to fieldwork, we compiled publicly available information on the turmeric supply chain to inform data collection. Where possible, interviewers were recruited locally from within or adjacent to the communities under study, and were extensively trained in social science research methods. Data collection instruments were designed as conceptual guides rather than rigid scripts, allowing interviewers to probe organically rather than adhering to verbatim question delivery. This method is in-line with semi-structured qualitative probing as described by Patton (2015).

A central component of interviewer training emphasized reframing the research objective away from individual attribution and toward the identification of broader industry practices. This approach was intended to reduce social desirability bias and encourage candid discussion of supply chain norms — recognizing that adulteration is a known phenomenon, and that the goal is to understand its mechanisms rather than assign blame.

Given the complexity of color adulteration across multiple supply chain stages, iterative probing over several months was used to establish precise and consistent terminology. Triangulation was employed throughout, drawing on multiple inference types to examine the same phenomena from different vantage points. This included interviews with diverse supply chain actors as well as regulatory stakeholders. Convergent accounts across sources were further verified against documentary evidence, including photographic documentation of adulteration processes.

Patton, M. Q. (2015). Qualitative research & evaluation methods: Integrating theory and practice (4th ed.). SAGE Publications.
